# Characterization of cytoskeletal and structural effects of INF2 variants causing glomerulopathy and neuropathy

**DOI:** 10.1038/s41598-023-38588-7

**Published:** 2023-07-25

**Authors:** Hiroko Ueda, Quynh Thuy Huong Tran, Linh Nguyen Truc Tran, Koichiro Higasa, Yoshiki Ikeda, Naoyuki Kondo, Masaki Hashiyada, Chika Sato, Yoshinori Sato, Akira Ashida, Saori Nishio, Yasunori Iwata, Hiroyuki Iida, Daisuke Matsuoka, Yoshihiko Hidaka, Kenji Fukui, Suzu Itami, Norihito Kawashita, Keisuke Sugimoto, Kandai Nozu, Motoshi Hattori, Hiroyasu Tsukaguchi

**Affiliations:** 1 Division of Nephrology, Second Department of Internal Medicine, Kansai Medical University, 2-5-1 Shinmachi, Hirakata, Osaka 573-1191 Japan; 2 Department of Genome Analysis, Institute of Biomedical Science, Kansai Medical University, Hirakata, Japan; 3 Department of Molecular Genetics, Kansai Medical University, Hirakata, Japan; 4 Department of Legal Medicine, Kansai Medical University, Hirakata, Japan; 5 Department of Gynecology and Obstetrics, Kansai Medical University, Hirakata, Japan; 6 Division of Nephrology, Department of Medicine, Showa University Fujigaoka Hospital, Yokohama, Kanagawa Japan; 7 Department of Pediatrics, Osaka Medical and Pharmaceutical University, Takatsuki, Japan; 8 Department of Rheumatology, Endocrinology and Nephrology, Faculty of Medicine and Graduate School of Medicine, Hokkaido University, Sapporo, Japan; 9 Department of Nephrology and Laboratory Medicine, Kanazawa University, Kanazawa, Japan; 10 Department of Internal Medicine, Toyama Prefectural Central Hospital, Toyama, Japan; 11 Department of Pediatrics, Shinshu University School of Medicine, Matsumoto, Japan; 12 Department of Biochemistry, Faculty of Medicine, Osaka Medical and Pharmaceutical University, Takatsuki, Japan; 13 Major in Science, Graduate School of Science and Engineering, Kindai University, Higashiosaka, Japan; 14 Department of Energy and Materials, Faculty of Science and Engineering, Kindai University, Higashiosaka, Japan; 15 Department of Pediatrics, Kindai University Faculty of Medicine, Osakasayama, Japan; 16 Department of Pediatrics, Kobe University Graduate School of Medicine, Kobe, Japan; 17 Department of Pediatric Nephrology, Tokyo Women’s Medical University, Tokyo, Japan; 18 Present Address: Toyama Transplantation Promotion Foundation, Toyama, Japan

**Keywords:** Nephrology, Neurology, Genetics, Clinical genetics, Medical genetics, Cytoskeleton, Mechanisms of disease, Organelles, Mitochondria

## Abstract

Focal segmental glomerulosclerosis (FSGS) is a common glomerular injury leading to end-stage renal disease. Monogenic FSGS is primarily ascribed to decreased podocyte integrity. Variants between residues 184 and 245 of *INF2*, an actin assembly factor, produce the monogenic FSGS phenotype. Meanwhile, variants between residues 57 and 184 cause a dual-faceted disease involving peripheral neurons and podocytes (Charcot–Marie–Tooth CMT/FSGS). To understand the molecular basis for *INF2* disorders, we compared structural and cytoskeletal effects of *INF2* variants classified into two subgroups: One (G73D, V108D) causes the CMT/FSGS phenotype, and the other (T161N, N202S) produces monogenic FSGS. Molecular dynamics analysis revealed that all *INF2* variants show distinct flexibility compared to the wild-type INF2 and could affect stability of an intramolecular interaction between their N- and C-terminal segments. Immunocytochemistry of cells expressing *INF2* variants showed fewer actin stress fibers, and disorganization of cytoplasmic microtubule arrays. Notably, CMT/FSGS variants caused more prominent changes in mitochondrial distribution and fragmentation than FSGS variants and these changes correlated with the severity of cytoskeletal disruption. Our results indicate that CMT/FSGS variants are associated with more severe global cellular defects caused by disrupted cytoskeleton-organelle interactions than are FSGS variants. Further study is needed to clarify tissue-specific pathways and/or cellular functions implicated in FSGS and CMT phenotypes

## Introduction

Focal segmental glomerulosclerosis (FSGS) is a clinicopathological condition characterized by a unique distribution of glomerular scarring that affects a part of the nephron (focal), and a portion of the capillary tufts (segmental)^1^. Patients with FSGS clinically display a steroid-resistant nephrotic syndrome (SRNS) and progressive deterioration of renal function. To date, over 60 genes have been reported to be associated with monogenic SRNS^2^. Most SRNS genes are expressed in glomerular podocytes, highlighting a key role of podocytes in maintaining filtration barrier function. Podocytes generate slit diaphragms between actin-enriched, foot processes and adapt to filtration stress by regulating changes in cell shape mediated by dynamic reorganization of actin networks^3^.

Mutations in the inverted formin 2 gene (*INF2*) are the most prevalent (12–17%) cause of autosomal dominant FSGS^4,5^. INF2 belongs to the diaphanous subfamily of formins, which act as actin assembly factors in a range of cellular functions, including cell migration, vesicle transport, organelle dynamics, and positioning^6^. *INF2* encodes a multi-domain protein comprising central formin homology 1 (FH1) and 2 (FH2) domains flanked by the N-terminal Diaphanous Inhibitory Domain (DID) and C-terminal Diaphanous Auto-regulatory Domain (DAD). INF2 has a unique ability to accelerate both polymerization and depolymerization of actin. The depolymerization activity is negatively inhibited through intra-molecular interplay between the DID and DAD^7^. DID mutations cause *INF2*-related disorders related to dysregulated autoinhibition that renders the INF2 molecule constitutively active. INF2 also interacts with the Rho family of small GTPases, other members of the formin family and cellular regulators to orchestrate organization of actin-based structures such as lamellipodia, filopodia, and stress fibers^6^.

*INF2* mutations were originally identified in dominant families having FSGS alone^8^. Over 60 disease-linked *INFs* mutations exclusively map to the DID encoded by *INF2* exons 2–4^9,10^. In rare, largely sporadic instances, other *INF2* mutations cause peripheral neuropathy associated with Charcot–Marie–Tooth disease (CMT-DIE, MIM#614455) in addition to FSGS^9^. *INF2* mutations causing FSGS alone or FSGS/CMT double phenotypes segregate in distinctive regions of the DID. Variants in the proximal half of the DID (residues Leu57 to Glu184) typically cause CMT with early-onset FSGS, whereas those locating in the distal half (residues Glu184 to Leu245) lead to late-onset, mild FSGS alone^10^. These observations raise the question of how these *INF2* mutations could distinctly impact two cell lineages, podocytes and Schwann cells. *INF2* mutations are associated with cytoskeletal disorganization and altered mitochondria dynamics, endosomal trafficking/targeting, and interactions with mDia, a formin protein having activity that is counterbalanced by INF2^8,9,11–14^.

However, the reason why cooccurrence of CMT with FSGS is linked to INF2-DID mutations, which exclusively cluster in the proximal region of the DID, is unclear.

To better understand the molecular basis of *INF2* disorders, we compared the structural and cellular effects of INF2 variants between two distinct subtypes: single FSGS and dual CMT/FSGS diseases, in our study cohort. Structural and molecular dynamics analysis revealed that all the mutations examined generally alter the molecular stability of intermolecular DID and DAD interactions. This alteration likely perturbs INF2 autoinhibition efficiency and/or frequency. However, the modeling revealed no apparent differences in structural dynamic characteristics between the two phenotypic subgroups. Immunocytochemistry of cells expressing INF2 variants enabled visualization of distinct cytoskeletal effects between the CMT/FSGS and FSGS subgroups. The CMT/FSGS variants had disorganized architecture of both actin and microtubules, which are the primary affected cellular components and are fundamentally the same as those for the FSGS variants. However, the CMT/FSGS variants cause more prominent cytoskeletal disorganization than the FSGS variants, and in turn have a more severe effect on mitochondria size, shape, and distribution.

## Results

### Clinical phenotypes of INF2 disorders

**Figure 1:**
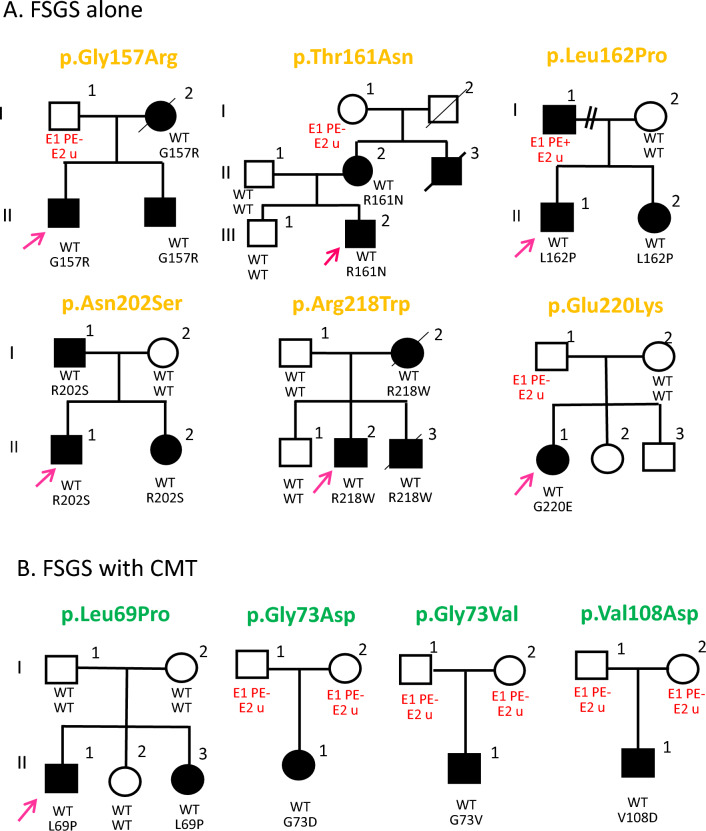
Pedigrees with *INF2* mutations showing dual CMT and FSGS or single FSGS phenotypes. Segregation of *INF2* variants is shown for each family. Squares and circles indicate male and female subjects, respectively. Slashes indicate deceased subjects and *arrows* indicate index patients. FSGS: Focal segmental glomerulosclerosis, CMT: Charcot–Marie–Tooth disease. E: examination, PE: physical examination, u, uninformative; WT: wild-type

We studied ten families, five of which followed a dominant transmission pattern (Fig. 1).

Clinical features of the 10 index patients with *INF2* mutations are summarized in Table 1. Six individuals exhibited FSGS alone, whereas the other five individuals had CMT and FSGS double phenotypes (CMT/FSGS). In accordance with previous reports^4,5,8,9^, the clinical manifestations of individuals with FSGS alone typically included proteinuria, ranging from moderate to nephrotic, that arose in early adolescence to adulthood. Some individuals exhibited microscopic hematuria and hypertension. Onset of proteinuria in the FSGS alone subgroup manifested at different times from childhood to adulthood (range 8–30 years-old, median age 16 years-old), and generally progressed to end-stage renal disease (ESRD) in the third to fourth decade of life (Supplementary Table S1). Notably, individuals with CMT/FSGS had proteinuria earlier (range 6 to 14 years-old, median 11 years-old) and progressed more rapidly to ESRD (range 12–17 years-old, median 15 years-old) than those with FSGS alone (range 13–36 years-old, median 24 years-old) (Supplementary Table S1, S2). Kaplan–Meier analysis confirmed that individuals in the CMT/FSGS subgroup manifested earlier onset of SRNS and progressed faster to ERSD (*P* < 0.01) (Supplementary Figure S1). Light microscopy of renal biopsy samples from the two subgroups showed a range of histology findings from minimal change to FSGS. The histology results could largely be subtyped into “FSGS, not otherwise specified (NOS)”^1^. In some patients with advanced disease, only diffuse global glomerular sclerosis was found. An immunofluorescence study revealed only non-specific IgM and C3 deposition with one exception: one index patient (case 4, II-1) was initially diagnosed as having IgA nephropathy. Electron microscopy showed podocyte foot-process effacement in the absence of electron dense depositions.

**Table 1:** INF2 variants in the 10 index patients with Charcot–Marie–Tooth disease and Focal Segmental Glomerulosclerosis.

Family	Exon	Amino Acid	Nucleotide Change		Inheritance	gnomAD	ClinVAR		dbSNP	CADD	Reported CaAR	References
		Change	cDNA position	Zygosity		MAF	Interpre-tation	Accession		Score	Max R %	
Single FSGS phenotype MIM613237												
1 AI	Exon 3	p.Gly157Arg	c.469G > C	Hetero	AD	0	NA	NA	NA	25.1	ND	NA
2 AJ	Exon 3	p.Thr161Asn	c.482C > A	Hetero	AD	0	NA	NA	NA-	23.6	ND	Tsukaguchi H, 2019
3 SH	Exon 3	p.Leu162Pro	c.485 T > C	Hetero	AD	0	LP	VCV001184454.2	NA	25.3	9	Caridi G, 2014
4 HO	Exon 4	p.Asn202Ser	c.605A > G	Hetero	AD	0	LP	VCV001697256.1	NA	23.4	49	Santin S, 2011
5 TO	Exon 4	p.Arg218Trp	c.652C > T	Hetero	AD	0	P	VCV000001052.5	rs267606878	25.4	0	Brown E, 2010
6 KI	Exon 4	p.Glu220Lys	c.658G > A	Hetero	de novo	0	P	VCV000523533.7	rs530391015	24.7	33	Brown E, 2010
Dual CMT/FSGS phenotype MIM614455												
7 OK	Exon 2	p.Leu69Pro	c.206 T > C	Hetero	mosaicism	0	VUS	VCV000637710.1	rs1595163820	26.9	0	Toyota K, 2013
8 NI	Exon 2	p.Gly73Asp	c.218G > A	Hetero	de novo	0	NA	NA	NA	26.5	0(Gly73Ser)	Hara M, 1984, Barua M, 2013
9 OS	Exon 2	p.Gly73Val	c.218G > T	Hetero	ND	0	P	VCV000472842.6	rs918089359	26.3	0(Gly73Ser)	Nagano C, 2020
10 YA	Exon 2	p.Val108Asp	c.323 T > A	Hetero	de novo	0	VUS	VCV000637711.1	rs1595164081	24.6	5	Toyota K, 2013

INF2 variants are annotated according to the nucleotide numbering of RefSeq NM_022489.4, where the A of the ATG-translation initiation codon is designated as position 1. Population allele frequency was obtained from the public database gnomAD (genome Aggregation Database); MAF, minor allele frequency; None of these variants were found in databases of Japanese healthy controls: HGVD, human genetic variation database; ToMMo, Integrative Japanese Genome Variation Database (iJGVD) of the Tohoku University Tohoku Medical Megabank Organization. AD, autosomal dominant. Scoring of deleteriousness was determined by Combined Annotation Dependent Depletion (CADD). In ClinVar annotations, variants are classified according to recommendation by the American College of Medical Genetics and Genomics (ACMG): P, Pathogenic; LP, Likely Pathogenic; VUS, Variant of uncertain significance. Calcium-dependent cellular actin organization (CaAR) was as described in a previous study^10^. CaAR measured in HeLa INF2 knock-out cells is shown as the percentage of cells with Max(R) values > 14.9. The values range from 100% (in control and cells expressing benign variants) to 0% (in cells expressing pathogenic variants). CMT, Charcot–Marie–Tooth Disease, FSGS, Focal segmental glomerulosclerosis, ND, not determined, NA, not applicable.

The five individuals with CMT/FSGS experienced difficulty walking with lower limb muscle wasting and weakness beginning at ages ranging from 10 to 15 years-old. The neurologic symptoms first manifested at ages similar to those for proteinuria (Supplementary Table S2). The peripheral neuropathy preferentially affected motor function in the distal lower extremities.

Electrophysiological studies revealed median-nerve conduction velocities (MCV) in the intermediate range (25–45 m per second)^15^ for two individuals, and the other three exhibited a predominant demyelinating feature MCV < 20 m per sec with no excitable profile or multilayered onion bulb formation. Hearing loss was noted in one case (case 10).

### Genetic analysis

**Figure 2:**
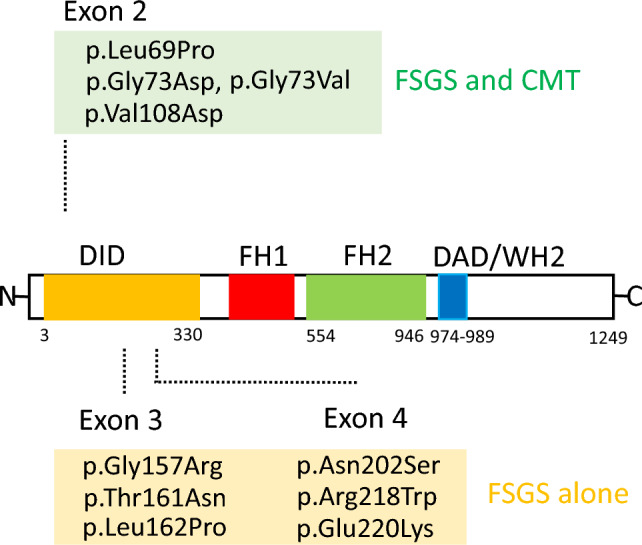
Locations of variants in the INF2 domain structure. The distribution of variants in INF2 functional domains is shown. Variants in exon 2 were found in individuals with a dual phenotype of Charcot–Marie–Tooth disease (CMT) and FSGS, whereas those in exon 3 and exon 4 were identified in patients with FSGS alone. INF2 is a large multi-domain protein composed of three functional domains: Diaphanous inhibitory domain (DID, orange), formin homology (FH) domains FH1 and FH2 (*red* and *green*), and diaphanous autoregulatory domain (DAD, *blue*). The DID interacts with the DAD within the same molecule (intramolecular autoinhibition) as well as with other actin regulators (e.g., small GTPase and mDia). The FH domains mediate both actin polymerization and depolymerization. Domain positions are according to UniprotKB-Q27J81. WH2: WASP homology 2; mDia: mammalian Diaphanous-related formin 1.

We found 10 heterozygous, missense INF2 variants in six familial and four sporadic FSGS individuals. All 10 variants localized to highly conserved residues in exons 2 to 4 that encodes the INF2 DID (Supplementary Figure S2). In silico analysis predicted that these mutations are certainly deleterious. Moreover, these variants were not present in 10,000 healthy ethnicity-matched control individuals (jMorp) or a large populations database containing over 246,000 chromosomes (gnomAD) (Table 1). Notably, mutations causing FSGS alone located within the distal half of DID. In contrast, those associated with the dual CMT/FSGS phenotype distribute in the proximal N-terminal half of DID (Fig. 2). The p.T161N and p.L162P mutations occurred in a border region that separates the mutation clusters of CMT/FSGS from the FSGS subgroup^5^.

### Structural modeling of INF2 variants

**Figure 3:**
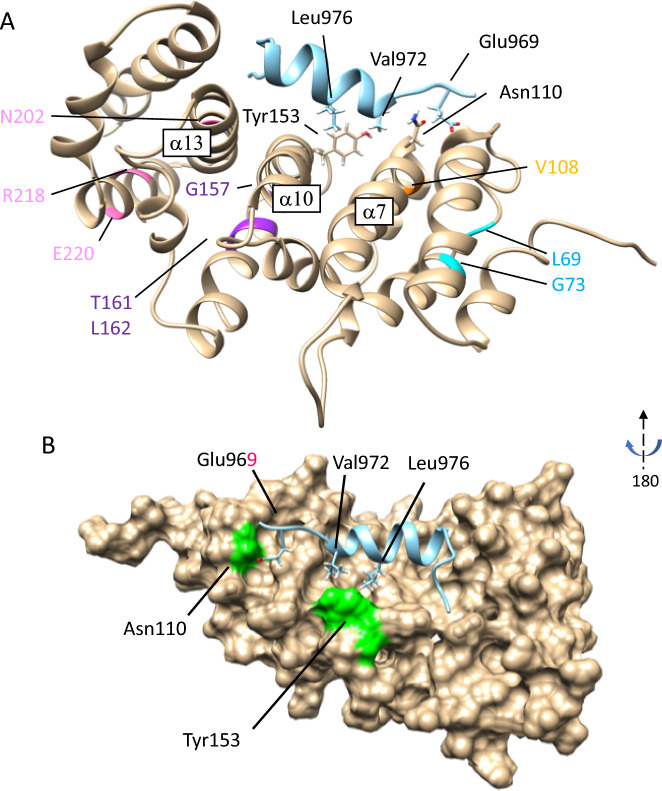
Structural modeling of human INF2. A 3D structure of human INF2 modeled on the mDia1 DID-DAD complex crystal structure (PDB 2F31)^16^ using the Rosetta program. (**A**) Locations for ten pathogenic variants in the DID are illustrated as ribbon diagrams; *Blue*: L69P, G73D (G73V), *Orange*: V108D, *Magenta*: G157R, T161N, L162P, *Pink*: N202S, R218W, E220K. Based on this model, the α helix of the N-terminal DAD (*turquois)* is predicted to contact the core pocket of DID. Side chains of selected residues involved in the DID/DAD interactions are shown in stick form: N110 and Y153 in DID, E969, V972, and L976 in the DAD. The central α helices, α7, α10, α13, from the 2nd, third, fourth armadillo repeats respectively, create the DID-binding pockets. (**B**) Core DID-DAD interaction. The DID is shown as a surface representation rotated horizontally by 180˚. The residues E969, V972, L976 in the N-terminal DAD are predicted to make direct contact with the surface-exposed partners N110, Y153 in the hydrophobic binding pocket of DID.

To evaluate the impacts of the mutations on the structure of human INF2, we first modeled the pathogenic variants in the DID using the crystal structure of the mouse mDia1 DID/DAD complex as a basis^16,17^. The side chains of hydrophobic residues of the DAD domain appeared to interact with the DID-binding pockets, which are formed by the α-helices α7, α10, and α13 of the armadillo repeats(ARM) (Fig. 3, Supplementary Figure S2, S3).This structural model was consistent with that derived from the deep learning-based AlphaFold2 program^18^ (Supplementary Figure S4). In this model, The CMT/FSGS variants mapped near the central core of the hydrophobic DAD-binding pocket, whereas the FSGS variants occurred in more external portions.

We next evaluated DID/DAD interactions in pathogenic INF2 variants by structure modeling. PDBsum interaction plots revealed that the DID-DAD interface of pathogenic INF2 variants has appreciable changes in both amino acid pairing and the number of hydrogen bonds relative to wild- type (Supplementary Figure S5). However, the interface area and solvent free energy (ΔG) did not significantly differ and does not seem to alter overall binding affinity of the DID/DAD interaction. (Supplementary Table S3)^19^. Modeling analyses suggest the structural effects of INF2 variants may be subtle changes in interacting pattern and the need of the evaluation for their molecular dynamics.

### RMSD analysis for INF2 variants

**Figure 4:**
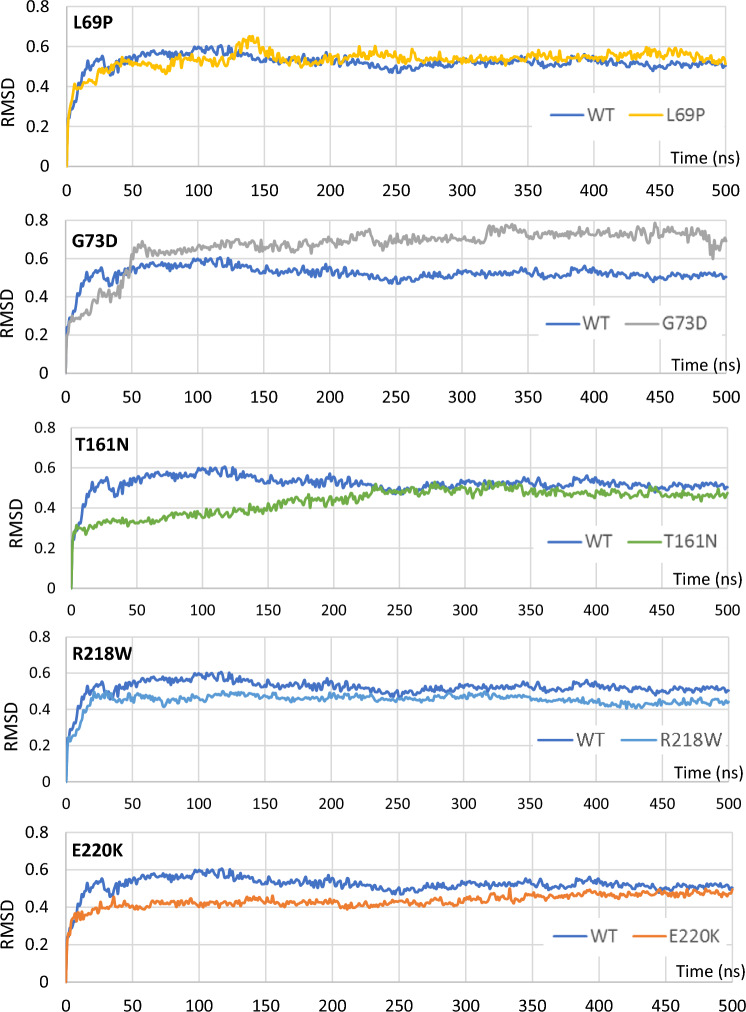
Comparison of molecular dynamics between INF2 wild-type and variants by Root-mean square deviation (RMSD) plots. RMSD values (nm) are plotted over the 500 ns (ns) MD simulations assuming an aqueous solution. A pair-wise comparison of the trajectories between the wild-type INF2 and variants discloses that the disease-causing variants have distinct flexibility of the DID-DAD autoinhibitory complex compared to the wild-type INF2 (WT). The RMSD of the WT increases over the initial 50 to 100 ns up to 0.5 nm and reaches a plateau till the end of simulation time period 500 ns. At the steady state, G73D variant has higher molecular dynamics, while L69P shows a minimal-change comparable to the WT. In contrast, the other three variants (T161N, R218W and E220K) display subtle decrease in RMSD than the WT.

We next investigated the effects of *INF2* missense mutations on dynamic flexibility and stability of the DID-DAD complex. The molecular dynamics of INF2 proteins was compared between the wild-type and five pathogenic variants by the comparative modeling using the Rosetta program and molecular dynamics (MD) simulation using the GROMACS program. We first accessed overall structural stability of the wild-type DID-DAD complex by analyzing the root-mean square deviation (RMSD) trajectories of their backbone atoms during the 500 ns simulation. The RMSD increased over the initial 50 to 100 ns up to around 0.5 nm and reached a plateau till the end of simulation time period 500 ns (Fig. 4). Five INF2 variants displayed the RMSD trajectories that got equilibrated between the 50 ns to 100 ns period similar to the wild-type, with the exception of T161N showing a gradual increase of RMSD that get stabilized around 200 ns. When the trajectories were compared at the steady state, the variants exhibited a variable degree of changes in overall average RMSD: G73D variant showed the largest average RMSD value (0.71 nm vs wild-type 0.52 nm), while L69P exhibited minimally increased level compared with the wild-type (0.55 nm) (Supplementary Table S4). In contrast, the other three (T161N, R218W, E220K) displayed subtle decrease in RMSD (0.44–0.46 nm) in relative to the wild-type. The variation in global RMSD of the INF2 variants suggest that the conformational changes may take place in the protein complex throughout the simulation.

### RMSF analysis for the INF2 variants

**Figure 5:**
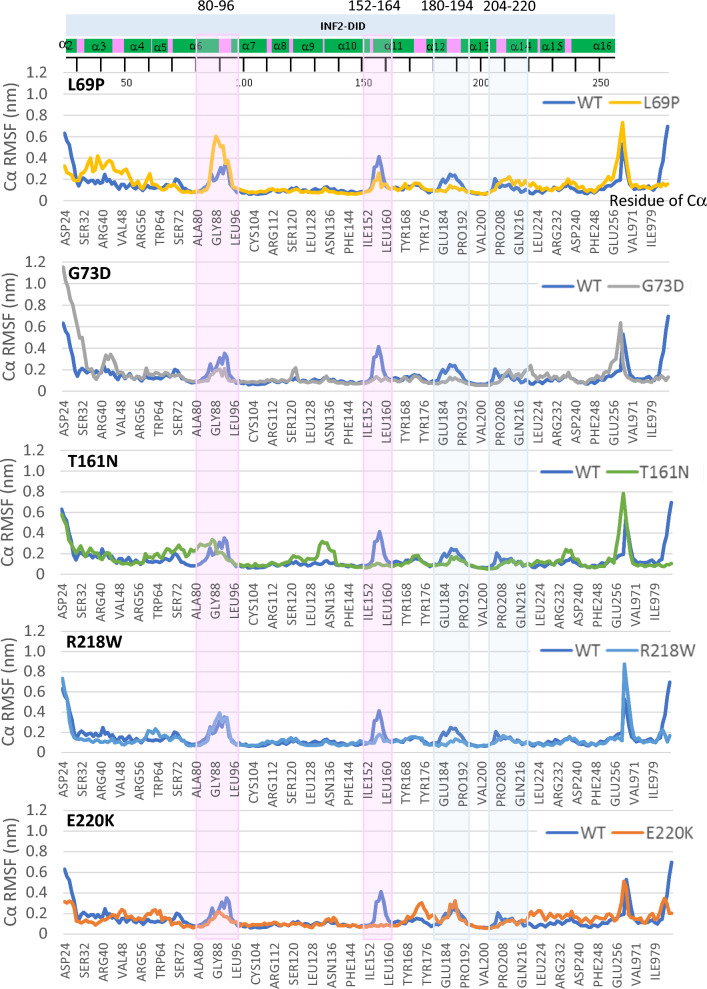
Comparison of molecular dynamics between INF2 wild-type and variants by Root-mean-square fluctuation (RMSF) plots. RMSF scores are shown by per residue of INF2-DID domain based on the Cα MD simulations of during 500 ns, assuming an aqueous solution. We used the simulation trajectories of the last 400 ns, the time interval at which the structure has become stable. A pair-wise comparison of trajectories between the wild-type INF2 (WT) and pathogenic variants displays the altered residual fluctuations in the DID domain. The highest (80–96, 152–164) and moderate-high fluctuating regions (180–194, 204–220) in the WT and pathogenic variants are highlighted with a light-pink and light-blue-shaded background, respectively. The first peak of fluctuation at residues 80–96 is increased (L69P), is broaden with split (T161N), is decreased (G73D, E220K), and is unchanged (R218W). The second peak of fluctuation at residues 152–164 is lost for four variants (G73D, T161N, R218W, E220K), while being preserved for L69P. The consensus secondary structure is shown schematically with α helices (green) and loops (pink).

To gain more insights behind the altered stability/flexibility of the DID-DAD complex, we estimated fluctuation of the individual amino acid residues within the complexes by measuring root-mean-square fluctuation (RMSF) for the backbone atoms of wild-type INF2 and variants. RMSF estimates the average deviation of each residue from the reference position within the staring minimized structures over the time of MD simulation. RMSF analysis of the wild-type INF2 DID-DAD complex revealed noticeable fluctuations in the four clusters: two highest fluctuating regions encompassing residue 80–96, and 152–164 (> 0.3 nm deviation from the reference positions), while the other two moderately fluctuating regions at residue 180–194, and 208–220 (around 0.1–0.2 nm) (Fig. 5). These four clusters with higher RMSF correspond to the loop segments connecting α helices of DID domain, the conformation of which is reported to be critical for interaction with the DAD domain (Supplementary Figure S2—S6)^16^.

Comparison of RMSF data between wild-type INF2 and variants revealed variations in residue dynamics in the DID domain for the pathogenic variants. First, in the first cluster residues 80–96, L69P variant showed increased amplitude, while T161N displayed broadening at the peak of fluctuations. The other two variants (G73D and E220K) showed mild decreased amplitude of fluctuation. The remaining one (R218W) exhibited subtle or indistinguishable differences for this peak. Second, in the second cluster residues 152–160, all the variants (G73D, T161N, R218W, E220K) lost the normal fluctuation peak, with exception for the L69P variant preserving the flexibility in this region. Our observations indicate that the pathogenic *INF2* mutations could cause variable changes in conformational flexibility of the DID domain.

### Flexibility of Residues at the DID-DAD interface

**Figure 6:**
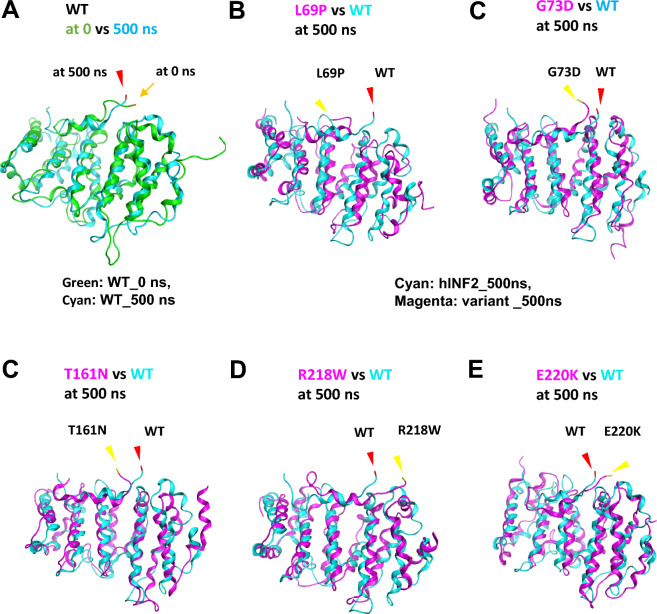
Comparison of the residue mobility at the DID-DAD interface between wild-type INF2 and five pathogenic variants. Molecular dynamics of human INF2 structures are simulated using GROMACS program, assuming that the protein is solvated in water with salt. Images of dynamic structures are captured with VMD (http://www.ks.uiuc.edu/Research/vmd/) and are compared between wild-type INF2 (WT) with pathogenic variants. The DAD residue Glu968, which is involved in the DID-DAD interaction, is indicated as an index tracking marker (arrow, at *t* = 0 ns; arrowheads, at *t* = 500 ns). (**A**) Mobility of Glu968 residue in wild-type INF2. Superimposed images at 0 ns (green) and 500 ns (cyan) are shown. The position of Glu968 at 500 ns (arrowhead, red) deviates from that of the reference at time 0 ns (arrow yellow), indicating that the Glu 968 residue of wild-type INF2 is mobile during the 500 ns-MD simulation. (**B**–**E**) Mobility of Glu968 residue in pathogenic INF2 variants. Dynamic structures of wild-type (cyan) and INF2 variants (L69P, G73D, T161N, R218W, E220K, magenta) are simulated at 500 ns and the superimposed images are shown. For all five pathogenic INF2 variants, the positions of Glu968 at 500 ns (arrowhead, yellow) are displaced from the reference location of WT (arrowhead, red), indicating that the pathogenic variants have the altered mobility at the DID-DAD interface compared to the WT.

We next evaluated the residue interactions at the DID-DAD interface of INF2 variants. We first compared the flexibility of Glu968 residue at the DID-DAD interface by overlaying two snapshots from the dynamic INF2 structure simulated at 0 ns and 500 ns, the latter of which represented the last frame of trajectories reaching their steady state of dynamics. The superimposed images showed that the Glu968 residues at the interface have a variable mobility among the pathogenic variants, compared with the wild-type (Fig. 6).

We then assessed the changes in dynamics of the residue pair at the DID-DAD interface. We first compared the time evolution of distance between sidechain of the backbone Cα atoms in Gly62 and Glu968 at the DID-DAD interface (Supplementary Figure S7). The residue pair distances were traced during the 500 ns simulation, a long enough to attain a stable conformation. The time evolution graph revealed that pathogenic variants fall into three classes with regards to their trajectories: One variant L69P showed the longest average inter-residue distance between Gly62 and Glu968 than the wild-type (1.32 vs 0.67 nm, Supplementary Table S4), while the other two (G73D, E220K) exhibited equal or smaller distance compared to the wild-type (0.65 and 0.52 nm, respectively). The remaining two T161N and R218W showed an intermediate distance level that fluctuates over time (average 0.70, 0.73 nm respectively).

We next compared the variation in residue contacts at the DID-DAD interface in total 500 simulated structures captured every 1 ns during the 500 ns-MD simulation between the wild-type and INF2 variants by measuring the dissociation frequency. Dissociation is defined as the distance between the sidechains of Gly62 and Glu968 deviates more than 3–5 Å from the reference position (at 0 ns) during the 500 ns simulation. L69P variant showed the most frequent dissociation (77% vs 13% for wild-type), while G73D had conversely the lowest value (0%). T161N and R218W exhibited a mild to moderate higher dissociation rate (37%, and 19% respectively), and E220K displayed slightly lower frequency (5%) compared with the wild-type (13%) (Supplementary Figure S8, S9).

Collectively, our MD simulations of the pathogenic variants reveal variable changes in average inter-residue distance and their fluctuations at the DID-DAD interface of the autoinhibitory complex. The observations suggest that mutations could perturbate native residual contacts, leading to the overall less stable structure.

### INF2 variants alter organelle morphology and actin network

We next assessed the cellular effects of INF2 variants by examining the subcellular distribution of INF2 proteins and actin networks in HeLa and COS-7 cells transfected with cMyc-tagged INF2 variants^20^ (Supplementary Figure S10). In HeLa cells, wild-type INF2 had a diffuse localization throughout the cytoplasm with a fine reticular, ER-localized pattern and perinuclear Golgi accumulation^8,21^. Actin stress fibers formed a thick parallel array that spanned the cells and was similar to that in un-transfected cells^22,23^.

**Figure 7:**
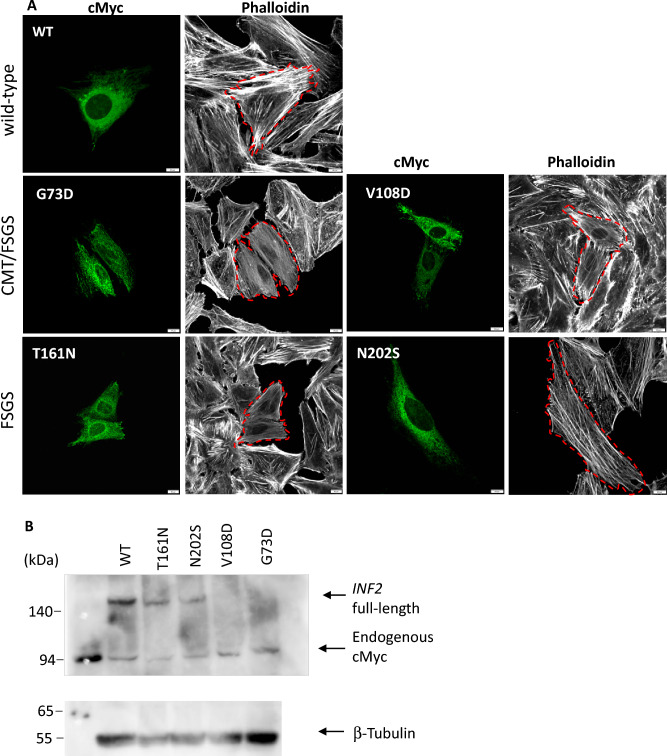
Expression of INF2 variants causing either single FSGS or dual CMT-FSGS phenotypes in HeLa cells (**A**) Subcellular localization of INF2 variants and actin network. cMyc-tagged wild-type INF2 and pathogenic variants were transfected into HeLa cells. At 8 h post-transfection, the cells were fixed and stained with anti-cMyc antibody (*green*) and phalloidin (*white*). Two variants (G73D, V108D) cause a dual CMT/FSGS phenotype, whereas the other two (T161N, N202S) lead to FSGS alone. The number of actin stress filaments was markedly reduced in cells expressing the G73D, V108D variant compared to those expressing the T161N, N202S variant. *Dashed lines* highlight the cell outline. Bars = 10 μm. (**B**) Western blot analysis of INF2 variants. INF2 protein expression in HeLa cells expressing cMyc-tagged wild-type INF2 or the G73D, V108D or T161N and N202S variants were analyzed by western blot. FSGS variant proteins (T161N, N202S) were less abundant than wild-type protein. CMT/FSGS variants (V108D, G73D) were barely detectable, suggesting that this variant may be more vulnerable to degradation. After the stripping step, internal control of β-tubulin was re-probed on the identical membrane with the same exposure time as the INF2 detection (5 min). Original blots/gels are presented in Supplementary Figure S14.

**Figure 8:**
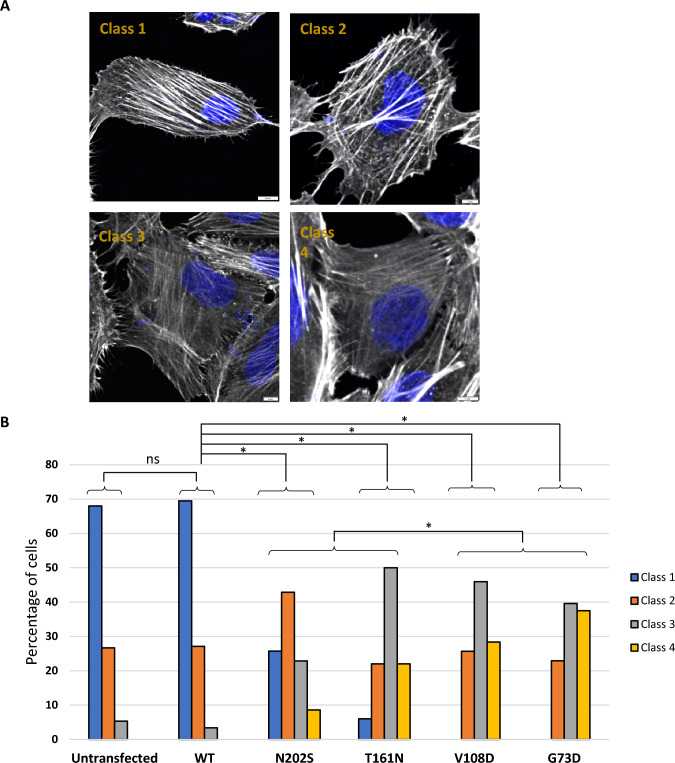
INF2 variants alter actin networks in HeLa cells expressing wild-type or pathogenic INF2 variants. (**A**) Representative images of four subgroups of actin stress fiber patterns. Confocal images were subdivided according to previous study^22,23^. Class 1: heavy, distinct cables cross > 90% of the central area of the cell; Class 2: at least two heavy, distinct cables enter the central half of the cell and the remaining area is filled with fine cables; Class 3: cells have only fine cables; and Class 4: no cables are detectable in the central area. Scale bars: 10 µm. (**B**) Histogram showing a proportional distribution of F-actin phenotypes. Immunofluorescence images (*n* = 100) were visually inspected and scored by two independent investigators. The proportional distributions of actin phenotype categories were significantly different between wild-type INF2 (WT) and each variant, and between FSGS- and CMT/FSGS-causing variant subgroups. Inter-subgroup differences were analyzed by Fisher exact test with multiple testing correction: not significant (ns) or significant (asterisks).

In contrast, HeLa cells expressing CMT/FSGS variants (G73D, V108D) exhibited an altered INF2 subcellular expression that was unevenly distributed in a coarse granular pattern having fewer perinuclear Golgi clusters (Fig. 7). Notably, HeLa cells expressing CMT/FSGS variants (G73D, V108D) were occasionally elongated along the long axis, reminiscent of cells expressing constitutively active mDia1^20^. In these fusiform-shaped cells, some INF2 puncta accumulated at the peripheral pole (Fig. 7). Quantitative analysis showed that cells expressing CMT/FSGS variants have significantly fewer actin stress fiber filaments relative to cells expressing wild-type INF2 (Fig. 8). Transfected COS-7 cells expressing CMT/FSGS variants also showed an aberrant INF2 subcellular distribution with Golgi dispersal resembling that of transfected HeLa cells and occasionally exhibited prominent cell spreading (Supplementary Figure S11). Transfection with INF2 in mouse podocytes showed subcellular distribution and actin network, essentially similar to HeLa and COS-7 cells (Supplementary Fig S12).

**Figure 9:**
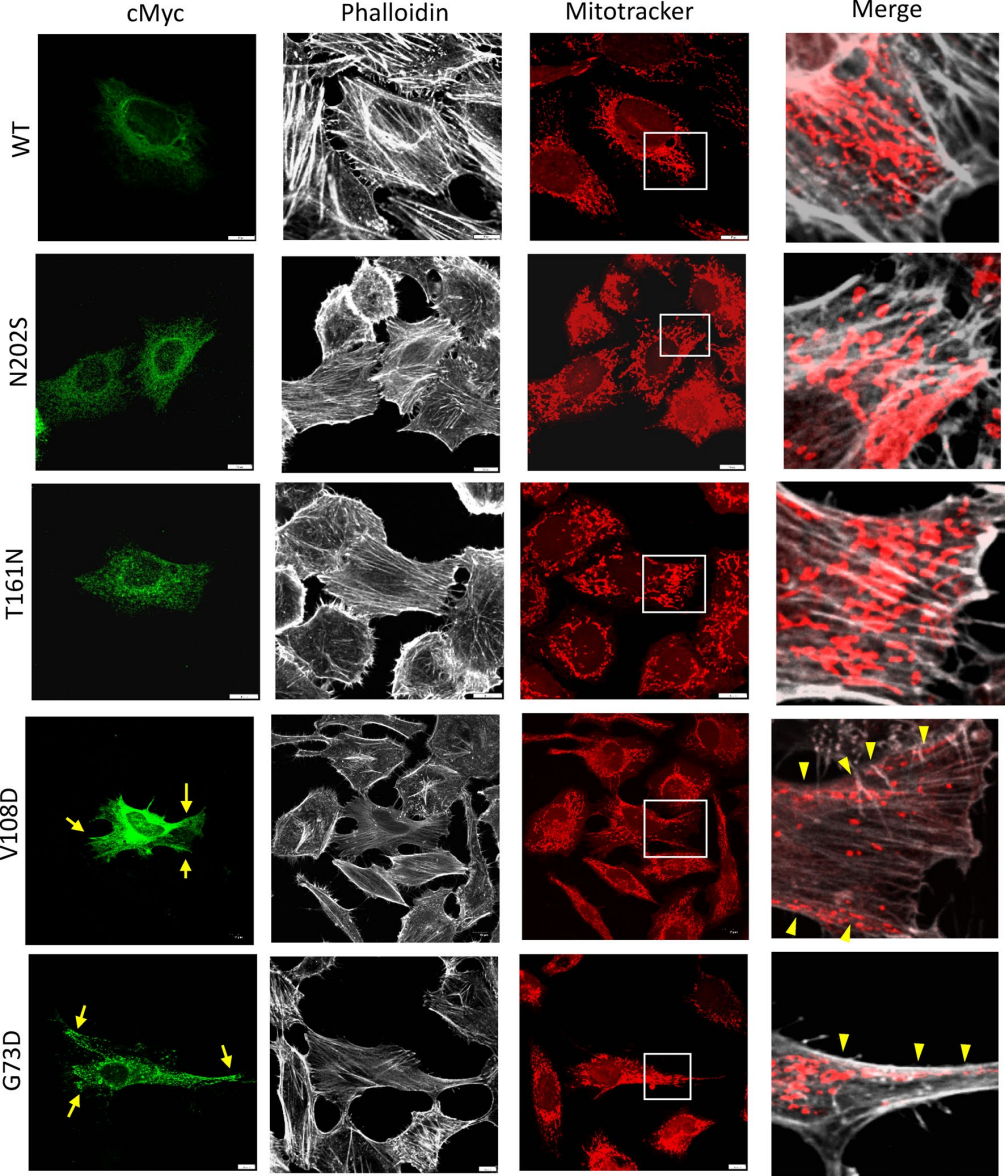
INF2 subcellular localization and distribution of mitochondria and actin in HeLa cells expressing wild-type or INF2 variants. Cells were labeled with MitoTracker 24 h after transfection with cMyc-tagged INF2 constructs, and then fixed with 4% paraformaldehyde. Cells were triple-stained with c-Myc antibody (Alexa 488), phalloidin (Alexa 647) and MitoTracker (Alexa 555). Cells expressing the wild-type and FSGS variant (T161N, N202S) showed a diffuse, fine reticular ER pattern with Golgi clusters, whereas those expressing CMT variant (G73D, V108D) exhibited a coarse punctate pattern with peripheral accumulation (*arrows*). The reduction in the number of actin stress fibers was more pronounced in cell expressing CMT/FSGS variants compared to those expressing FSGS variants. In cells expressing wild-type INF2, mitochondria were evenly distributed in the perinuclear region. In contrast, all pathogenic variants caused mitochondria to mislocalize to the cell periphery (*arrowheads*). The extent of mislocalization was more severe in cells expressing CMT/FSGS variants compared to those expressing FSGS variants. Cells expressing the T161N variant displayed an intermediate configuration, with an almost equal distribution of perinuclear and peripheral patterns. Scale bars: 10 μm.

HeLa cells expressing variants associated with FSGS alone (T161N, N202S) retained the diffuse ER pattern with Golgi accumulation similar to wild-type INF2. However, these cells had fewer actin stress fibers than those expressing wild-type INF2, indicating that FSGS variants have a cytoskeletal dysregulation that is between that seen for the wild-type and CMT/FSGS variants (Figs. 7, 8 and 9). The severity of actin disorganization in cells expressing the FSGS INF2 variant was further verified by automated-image classification using the deep learning tool ALEXNET. After training the neural network on a manually-labeled representative dataset (normal *vs*. fewer cables), the test images were automatically processed and classified into the two categories. Cells expressing T161N were principally classified into the “fewer stress fiber” category, while those expressing wild-type INF2 were overall judged as having a normal pattern (*p* < 0,001, Supplementary Figure S13).

Western blot analysis of lysates from HeLa cells expressing wild-type INF2 revealed a single major band having an apparent molecular mass of 170 kDa. The proteins were less abundant for FSGS-causing variants (T161N, N202S) than for the wild-type. Moreover, in cells expressing the variants associated with CMT/FSGS (G73D, V108D) the INF2 protein levels were barely detectable, suggesting that these variant proteins, particularly of the CMT/FSGS subtype, were more labile and thus more susceptible to degradation than wild-type (Fig. 7B, Supplementary Figure S14). Taken together, cells expressing CMT/FSGS variants displayed more prominent alterations in actin organization, cell shape, and organelle arrangement (i.e., altered ER pattern with Golgi dispersal) than FSGS variants, which likely reflects the more pronounced defects in their subcellular distribution as well as the changes in INF2 protein stability.

### INF2 variants alter mitochondria morphology and distribution

We next examined mitochondria morphology and distribution in fixed HeLa cells expressing c- Myc tagged INF2. Cells expressing FSGS-type variants (N202S) had mitochondria that were mostly distributed in the perinuclear region in a pattern similar to that seen for cells expressing wild-type INF2 (Fig. 9). In contrast, cells expressing CMT/FSGS variants (G73D and V108D) displayed a prominent change in mitochondria distribution, with a tendency toward peripheral localization with longitudinal, parallel alignment of some mitochondria along the disorganized actin filaments (Fig. 9). Cells expressing the T161N variant exhibited an intermediate phenotype in terms of shape and subcellular distribution in which mitochondria had a predominantly perinuclear localization with some aberrant enrichment at the cell periphery (Fig. 9).

**Figure 10:**
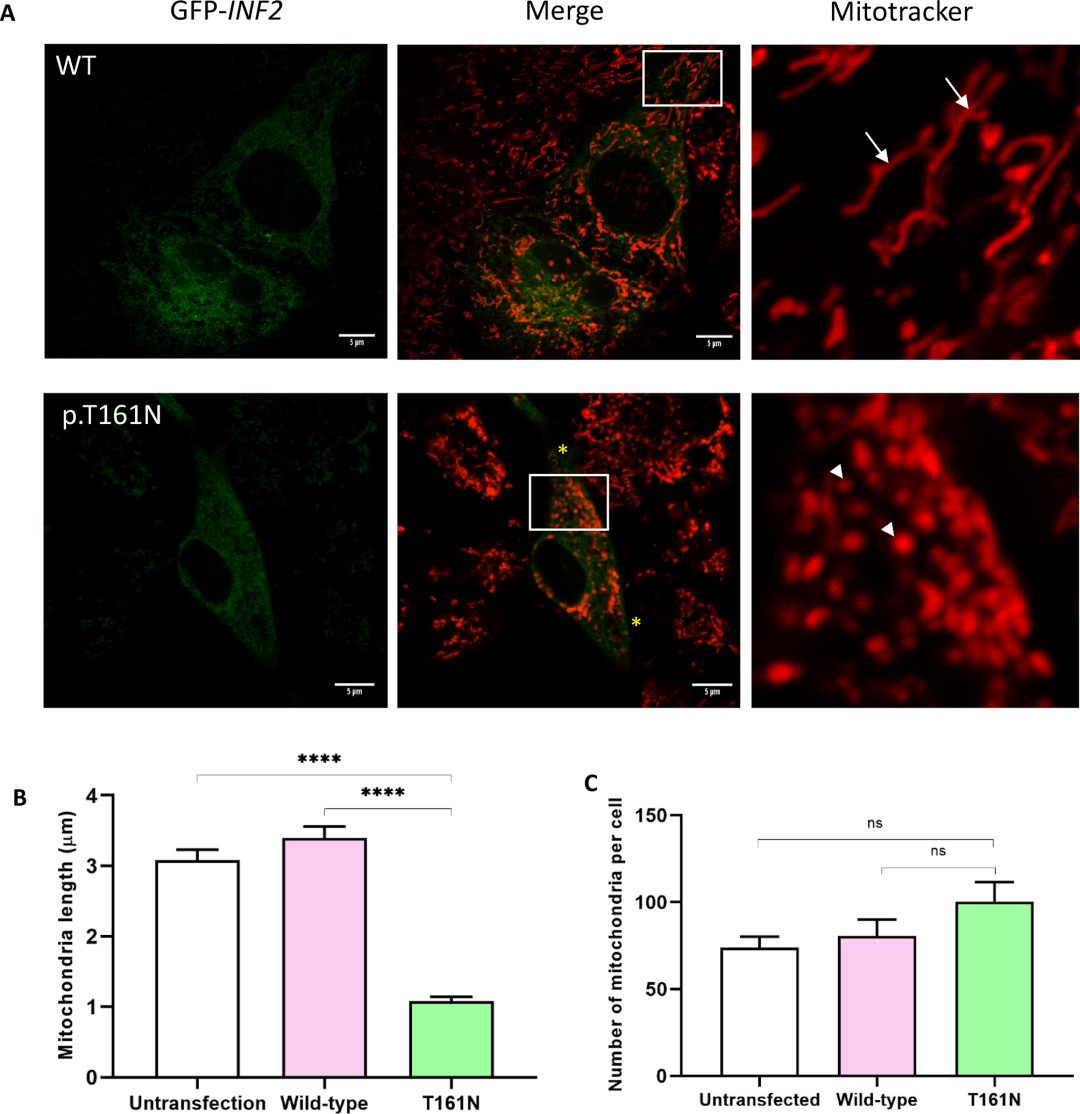
Mitochondria morphology in HeLa cells expressing either wild-type or T161N INF2 variant. (**A**) Mitochondria morphology in living cells. Cells were labeled with MitoTracker 8 h after transfection with GFP-tagged wild-type *INF2* or the T161N variant. Images were captured using filters at 488 nm (GFP, green) and 555 nm (MitoTracker, red). Mitochondria were more fragmented in cells expressing the T161N variant (*arrowheads*), and showed a mostly tubular pattern compared to cells expressing wild-type (*Arrows*). *Asterisks* indicate misdistribution at the cell periphery. Scale bars: 5 µm. (**B**) Quantitation of mitochondrial length. The length of 10 mitochondria per randomly-chosen area in the cytoplasm was measured using ImageJ (*n* = 20 areas for each group). Cells expressing the T161N INF2 variant had a 2.76-fold reduction in mitochondria length. Data are shown as mean ± SEM. (**C**) Quantitation of mitochondria number per cell. The number of mitochondria was analyzed using the Count and Measure tool with the Cellsens program (n = 15 cells for each group). Cells expressing the T161N INF2 variant had significantly higher numbers of mitochondria than those expressing wild-type INF2. The number of mitochondria in the wild-type-transfected and untransfected control cells was non-significant difference *(p* = 0.06). Data are shown as mean ± SEM.

**Figure 11:**
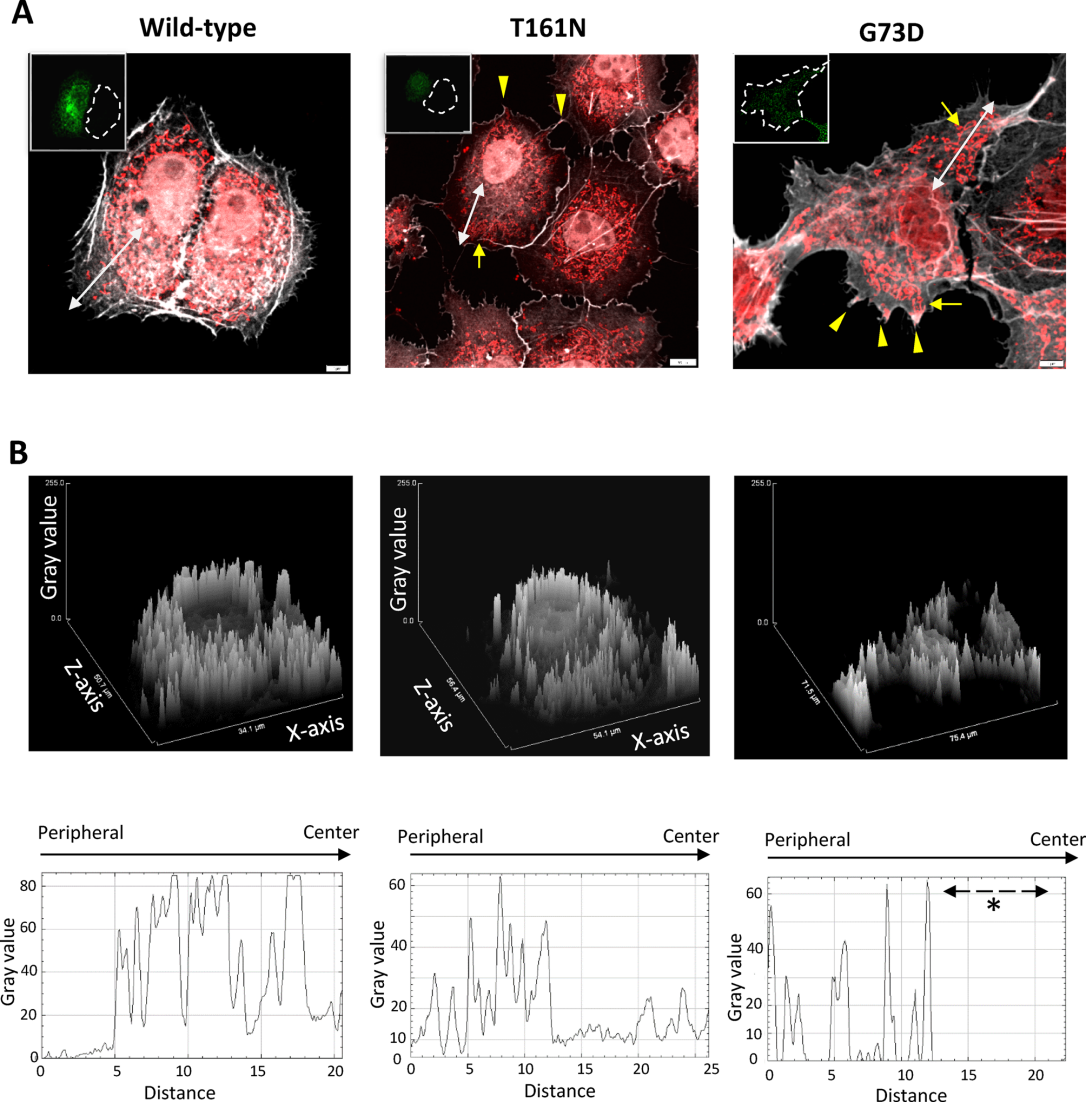
Mitochondria morphology and distribution in COS-7 cells expressing either wild-type or T161N INF2 variant. (**A**) Changes in mitochondria in INF2-expressing cells. cMyc-tagged wild-type INF2 or the T161N variant was transfected into COS-7 cells, which were fixed and labeled with anti c-Myc antibody (green) and MitoTracker (red) 8 h post-transfection. Cells expressing the T161N variant exhibited more frequent mitochondria fragmentation and misdistribution at the cell periphery (*arrows*); some accumulation was seen in cell processes (*arrowheads*). In contrast, cells expressing wild-type INF2 showed tubular structures that preferentially accumulated in the perinuclear region. Broken lines indicate the outline of untransfected cells. Scale bars: 10 μm. (**B**) Geometric profiling of mitochondria distribution in *INF2-*expressing cells. The intensity profiles of mitochondria along the white lines denoted in panel A were plotted with ImageJ. The Y-axis represents the intensity of the mitochondrial signals (gray value), whereas the X- and Z-axis represent cell axis and spreading, respectively. Areas marked by the white dotted line were analyzed. Cells expressing CMT/FSGS-type variants (G73D) displayed a prominent peripheral misdistribution of mitochondria; the lack of signals in the central area is indicated by the asterisk. Cells expressing T161N variants exhibited an intermediate pattern of misdistribution in which both normal perinuclear localization and occasional misdistribution to the cell periphery were present.

We next examined the mitochondria morphology in living cells transfected with INF2 variants using the T161N variant as a representative pathogenic variant and labeling of the mitochondrial network in live cells using MitoTracker. In cells expressing wild-type INF2, mitochondria had a normal tubular pattern with perinuclear accumulation. In contrast, expression of T161N variants altered the mitochondrial morphology from the normal tubular pattern into a globular pattern with a fragmented appearance (Fig. 10, Supplementary Figure S15, 16). Moreover, some mitochondria were aberrantly distributed at the cell periphery (Figs. 9, 11). Quantitative analysis indicated that the average mitochondria length in T161N-expressing cells decreased by a factor of 2.76 compared to wild-type, suggesting that this pathogenic variant increases mitochondria fragmentation (Fig. 10, Supplementary Figure S15, 16). These observations indicate that the INF2*-*T161N variant alters mitochondria size, shape, and distribution through cytoskeletal disorganization.

### INF2 variants alter microtubule and mitochondria organization

**Figures 12:**
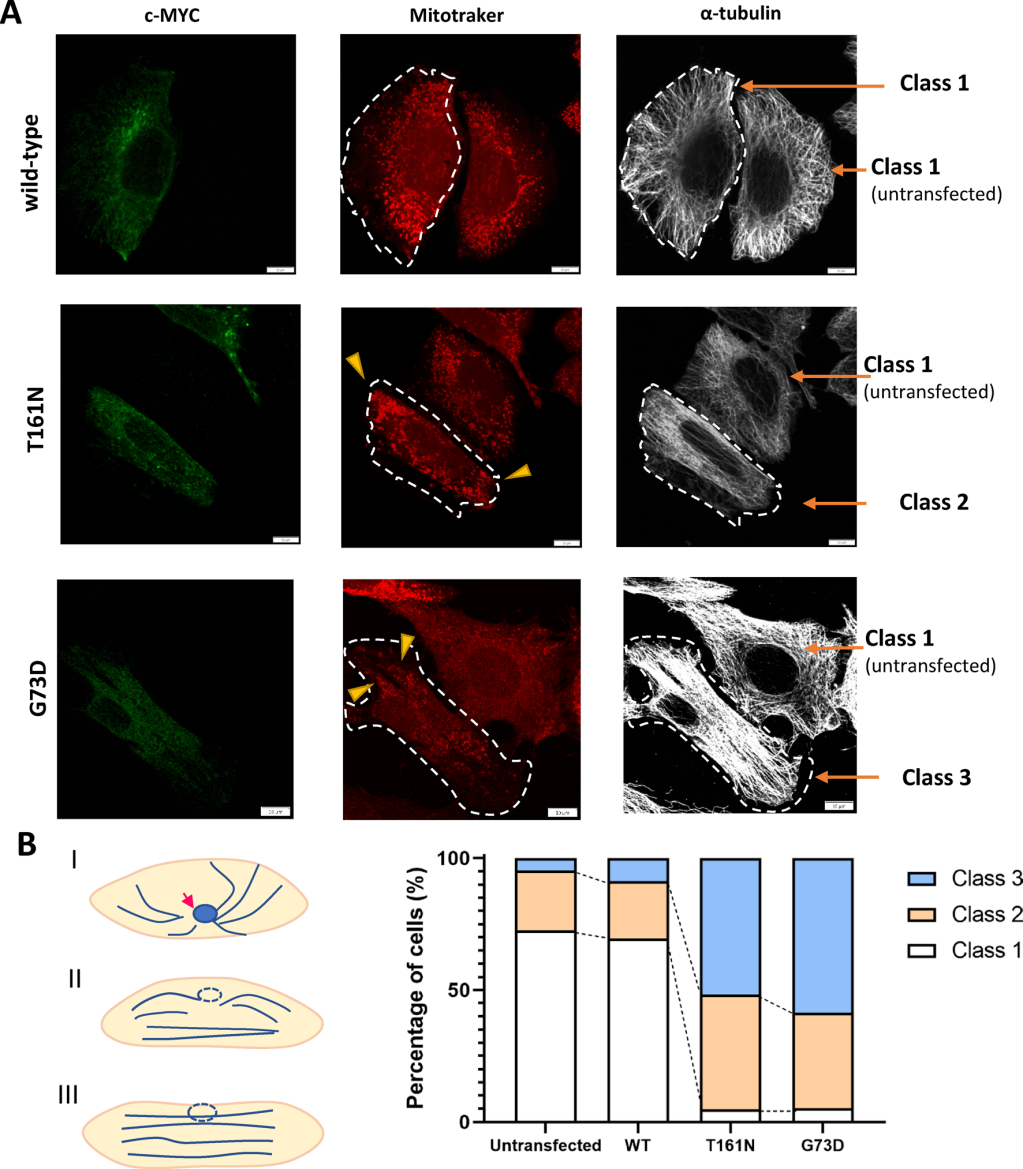
Microtubule array and mitochondria distribution in HeLa cells expressing *INF2* variants. HeLa cells transfected with eGFP-tagged wild-type INF2*,* T161N, and G73D variants were stained with MitoTracker (*red*) and anti-α-tubulin (*white*). The outline of the transfected cells is indicated by *dotted lines*. (**A**) Subcellular distribution of mitochondria and microtubules. Mitochondria in cells expressing the T161N variant (*arrowhead*) were mis-distributed to the cell periphery, while those in wild-type cells were predominantly located in the perinuclear region. In cells expressing wild-type INF2, microtubules were organized in a diffuse, radiating pattern. Cells expressing T161N and G73D variants had varying degrees of aberrant alignment and had parallel bundles in particular. Bars = 20 μm. (**B**) The three subtypes are classified by the following criteria: Class 1: normal subtype: oriented in an array radiating from the centrosome area near the nuclei (normal subtype). *Arrow* indicates microtubule organizing center. Class 2: intermediate subtype: some regions of the cytoplasm are occupied by microtubules arranged in parallel, while the remaining area is filled with a normal radiating array; and Class 3: disorganized subtype: microtubules are globally aligned in parallel (bundle formation) along the long axis of the cells. The diagram shows a proportional distribution of these phenotypes in HeLa cells. Cells from three independent transfections were classified (*n* = 200 cells).

We next compared the microtubule arrangement of HeLa cells expressing N-terminal GFP-tagged wild-type INF2 and pathogenic variants. In untransfected cells and those expressing wild-type INF2, microtubules generated a radial array that spread evenly throughout the cytoplasm (class 1, in Fig. 12). This pattern suggests normal nucleation and polymerization from a perinuclear, microtubule organizing center. Meanwhile, cells expressing pathogenic INF2 variants exhibited a disorganized microtubule network. Cells expressing the T161N variant showed an uneven, mixed pattern of microtubule disorganization in which the cortex was filled with longitudinally aligned, thick microtubule bundles and a sparse distribution of normal thin, radiating microtubule arrays (class 2). Cells expressing G73D variant typically exhibited global dysregulation in which most microtubules were disorganized as bundles aligned parallel to the cell long axis and lacking polarity (class 3).

Mitochondria double-labeling showed that, in sharp contrast to the predominant perinuclear localization seen in untransfected and cells expressing wild-type INF2, mitochondria in cells expressing the G73D or T161N INF2 variant had mitochondria that were mis-distributed to the cell periphery, suggesting defective mitochondria trafficking along microtubules.

Quantitative classification of the morphologic patterns into three subgroups revealed that approximately 70% of both wild-type and un-transfected cells exhibited randomly-radiating microtubules (class 1). In contrast, about 60% of cells expressing T161N variant exhibited a partially disorganized microtubule pattern (class 2), whereas about 70% of G73D variant-expressing cells displayed a global microtubule disarrangement (class 3, *n* = 200 cells for each group) (Fig. 12). The observations indicated that pathogenic INF2 variants promoted a disorganized microtubule architecture and mitochondrial distribution, and the CMT/FSGS variants cause more severe disorganization than the FSGS variants.

## Discussion

**Figure 13:**
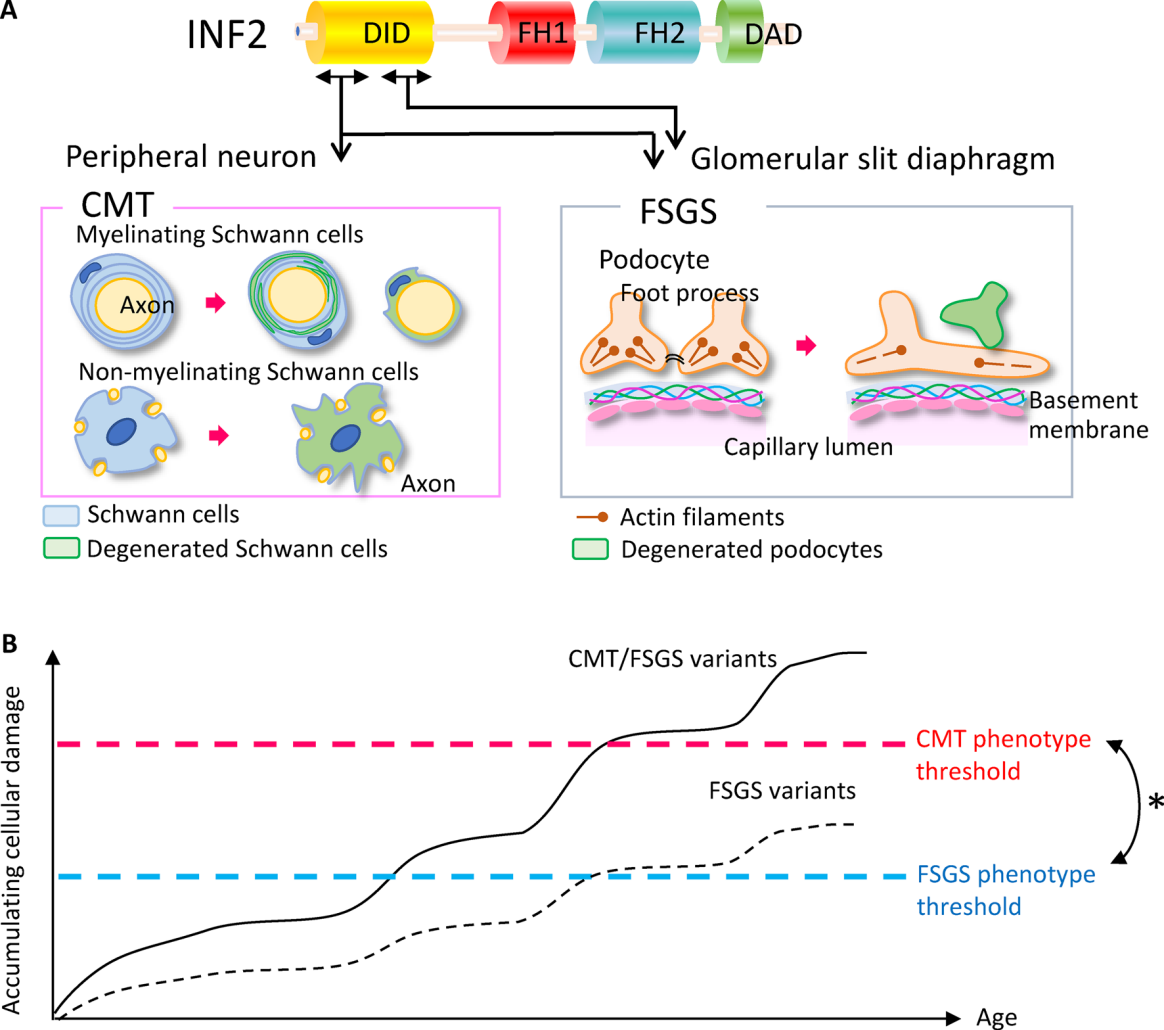
Hypothetical model of pathogenic mechanisms underlying a spectrum of* INF2* disorders. (**A**) Locations of *INF2* mutations and clinical phenotypes. Two INF2 disease phenotypes, dual CMT/FSGS or single FSGS, are linked with INF2 mutations, which segregate with distinctive regions of the DID. CMT/FSGS variants cluster in the residues 57 to 184 of the DID, whereas FSGS variants mostly locate in the residues 184 to 245 of the DID. Degenerated Schwann cells produce a thin myelin sheath (myelinating neuron) and/or supernumerary protrusions (non-myelinating neuron). Degenerated podocytes detach from the basement membrane. (**B**) Proposed model for disease pathogenesis. *INF2* mutations primarily disrupt actin and microtubule organization that subsequently leads to more global cellular dysfunction including defective mitochondrial morphology and vesicle trafficking. The mutational effects become more severe with age, reflecting a gradual accumulation of subtle damage. When cellular damage reaches a critical threshold, cell death occurs and disease phenotypes begin to manifest clinically. Our expression study shows that CMT/FSGS variants have more deleterious effects than FSGS variants in terms of cytoskeletal and organelle disorganization. Based on (1) only a few *INF2* mutations that cause CMT alone and (2) the fact that FSGS is usually severe if CMT phenotypes co-exist, it is plausible that podocytes are more susceptible to *INF2* mutation-induced injury than are Schwann cells, thereby supporting the hypothesis of a lower pathogenic threshold for FSGS than CMT. However, 3 in 30 reported cases with CMT/FSGS variants showed a predominant CMT phenotype (CMT alone or with minimal glomerulopathy). A larger mutational study is mandatory to verify our hypothesis that pathogenic threshold of CMT (*asterisks*) may generally exceed over that of FSGS. DID, Diaphanous inhibitory domain; DAD, Diaphanous autoregulatory domain; FSGS, Focal segmental glomerulosclerosis; CMT, Charcot–Marie–Tooth disease.

In the present study, we explored the molecular mechanisms by which *INF2*-DID mutations between residues 57 and 184 cause two subsets of disorders, CMT and FSGS. Structural modeling and molecular dynamics simulation revealed no consistent differences between the variants associated with the two distinct phenotypes. Our expression studies disclosed that both CMT/FSGS and FSGS variants share a common mode of action that principally affects actin-microtubule organization. Such cytoskeletal disorganization perturbs global cell characteristics such as the shape and position of organelles. Variants associated with CMT/FSGS have a more prominent cytoskeletal effect than variants associated with FSGS alone and thus in turn confer more severe mitochondria defects. In particular, mitochondrial defects are a critical consequence of cytoskeletal dysregulation in *INF2* disorders, given that such abnormalities are frequently observed for other clinical subtypes of CMT as well as in FSGS^2,3,19^ . Locations of our *INF2* mutations are consistent with the reported hot spots, where single FSGS-causing variants locate within the distal half of DID, whereas those for the dual CMT/FSGS phenotype distribute in the proximal N-terminal half of DID. However, recent catalogues of mutations have disclosed no consistent difference in the locations among the two clinical subtypes (Supplementary Table S5, S6). For example, there are considerable intra- and/or interfamilial variability of the *INF2* mutations. Moreover, patients with *INF2* variants affecting the proximal DID domain (residue Arg91, Val102, Cys104) have been reported to exhibit the CMT alone or CMT with minimal glomerulopathy^19^ (Supplementary Table S6). Thus, in a majority of the INF2 disorders, renal diseases are a common denominator in the clinical phenotype. However, further genotype–phenotype correlation study is necessary to verify the hypothesis that podocytes might be more susceptible to INF2-induced cytoskeletal aberrations than neurons^9,19^. In our study cohort, patients with the dual CMT/FSGS phenotype manifested the two diseases nearly simultaneously and progressed much faster to ESRD than those with FSGS alone^24^. These clinical observations suggest that patients with dual CMT/FSGS suffer from more severe, global cellular damage, which, like patients with FSGS, likely arises from a common mechanism that primarily affects cytoskeletal organization. Consistent with this possibility, results of expression studies confirmed that cells expressing CMT/FSGS variants indeed had more prominent cytoskeletal disarrangement and organelle perturbation compared to FSGS INF2 variants, and the disorder associated with the CMT/FSGS variant would exceed the critical threshold to trigger disease in both Schwann cells and podocytes (Fig. 13). However, a considerable phenotypic diversity has seen reported for the INF2 variants. Further study with a larger mutational survey is necessary to understand the genotype–phenotype correlation.

### Structural basis of INF2 disorders

Structural modeling revealed that the *INF2* mutations causing CMT/FSGS were exclusively located in the concave pocket of the DID that forms the DAD binding interface and is critical for self-autoinhibition^5,8,9^. Our structural studies supported that the *INF2*-DID mutations could impair DID- DAD interactions. First, the structural modeling of pathogenic INF2 variants showed changes in amino acid pairings and the number of hydrogen bonds, which are two key elements for the DID-DAD interaction. However, our estimation for the free energy showed no observable difference between CMT/FSGS and FSGS variants, in accordance with previous studies^19^. Second, our molecular dynamics simulations, assuming an aqueous solution, revealed that all the pathogenic *INF2* mutations could alter the flexibility and stability of the DID-DAD complex. We found that the mutations affect overall structural stability by RMSD analysis and cause variable changes in the residual dynamics by RMSF analysis. In order to estimate the impact of the mutations on the protein dynamics accurately, we performed the MD analysis for an adequate time (500 ns). Such MD simulations are long enough for the protein to explore conformational space and settle the most stable one. This allowed precise estimation of amino acid interactions, e.g., the changes in side-chain interaction over time (Supplementary Figs. S6, 7, 8, 9).

The MD analysis revealed a variation in the interacting residue flexibility among the pathogenic variants. Our RMSD and RMSF analyses disclosed a trend that CMT/FSGS variants may cause more pronounced changes in the flexibility of the DID-DAD complex, compared with the other INF2 variants (Figs. 4, 5 and 6, Supplementary Figure S6–S9). However, we could not find any consistent trends of MD that might explain the difference of phenotypic severity between severe dual CMT/FSGS and mild single FSGS disease. This is also partly because of our limited numbers of variants analyzed and variability in structural effects of mutations. Specifically, some local residue fluctuations may directly affect the conformation of binding pockets, while the others may secondarily influence the size and the curvature of the DAD recognition surface and its neighbors. For example, either increase (destabilized) or decrease (over-stabilized) in the residue flexibility of DID domain, could sterically hinder the local DID-DAD interaction, thereby affecting the binding affinity and/or frequency of the DID-DAD interfaces^25^.

We found a remarkable high RMSF for the top three clusters in the DAD-binding regions of the DID in wild-type INF2 at residues of 80–96, 152–164, and 180–194. Each region corresponds to the flexible loop structures connecting of α helices in the DID-binding pockets, which are assembled with the central helices (α7, α10 and α13) from the second, third, and fourth armadillo repeats, respectively (Supplementary Figure S3, S6)^16,25^. Those fluctuating loci are located in the vicinity of interacting residues, 107–115, 146–152, and 203–206, those of which are predicted to form the DAD-interacting pocket ^16,25^. The super-helical structures formed by armadillo repeats determines the curvature of the protein that suits for the peptide binding. Numerous studies show that armadillo repeats formed by DID α helices allow these proteins to have various interaction partners and functions in the cell and are conserved across eukaryotic kingdom^16,25^. Our results together with others indicate that the DID mutations could alter the native interaction pattern of neighboring residues in the vicinity of mutations, thereby compromising the protein stability and flexibility of the DID-DAD autoinhibitory complex. Further MD simulation, *e.g.* RMSF determination including more pathogenic variants and comparison with benign variants, will help understand which individual residues might play a key role in the protein complex structural fluctuations.

### General molecular basis for INF2 disorders

We sought to determine how CMT/FSGS variants could be more biologically damaging than FSGS variants. The transfection protocols for INF2 variants were optimized to minimize the cytotoxicity by parallelly monitoring the cellular phenotype of appropriate controls. We compared the cellular effects of INF2 variants, under the conditions where cells transfected with a mock vector or wild-type INF2 cDNA (plus the surrounding untransfected cells) did not show any harmful alterations in actin-microtubule organization and organelle interaction (Supplementary Figure S10). The deleterious effects of the CMT/FSGS variants might be due to their reduced INF2 dosage as was suggested by results of western blotting showing that protein levels were decreased for the CMT/FSGS variants relative to wild-type. Such a trace amount of the CMT/FSGS variants could even trigger disease^10^.

Alternatively, CMT/FSGS variants affecting the residue 57 to 184 of DID could impair preexisting molecular interactions or pathways specific to neuronal tissues^10^. For example, CMT/FSGS variants may preferentially interfere with acute cell adaptation in response to stress^10^. Previous studies showed that INF2 is indeed involved in remodeling the actin architecture during tissue repair after injury^10,11^. *INF2* mutations causing CMT/FSGS may thus render the affected tissue more susceptible to injury due to delays in reparative processes needed to recover from injury. Previous studies revealed that pathogenic INF2 variants disrupted calcium-induced, transient actin bursts in living cells and could alter long-standing transcriptomes regulated by serum response factor that in turn dysregulate other cell functions beyond the cytoskeleton^11,26^ Further study is needed to define the tissue-specific mediators that act in podocytes and/or Schwann cells^9^.

### Microtubule disorganization in INF2 disorders

We found that the extent of microtubule network disruption is proportional to the severity of actin disorganization. INF2 is known to colocalize, directly bind, and mediate both bundling and stabilization of microtubules^27–29^. Therefore, two mechanisms for the deleterious effects of FSGS/CMT variants are plausible: *INF2* mutations could disorganize microtubule networks through direct effects on microtubules and/or have indirect consequences arising from altered actin- microtubule interactions. Here we found that HeLa cells expressing pathogenic INF2 variants tended to have fusiform cell shapes with bipolar elongation (Fig. 7, 9), as well as an unusual parallel arrangement of microtubules. These cell features are reminiscent of cells expressing constitutively active mDia1 variants^14,20^, in which alterations in microtubules primarily drive cell elongation. In contrast, previous studies with *INF2* knock-out cells demonstrated that INF2 plays a key role in lamellipodia formation of the cell cortex^30^. Since a range of microtubule crosstalk with actin-based structures in the cell cortex is essential to stabilize microtubules^27,29^, we speculate that in cells expressing INF2 variants, microtubules needed to capture cortical actin may be defective. The mechanisms likely involve capping of faster-growing plus-end microtubules through interaction with different formins and other “stabilisome” proteins comprising plus-end-tracking proteins and scaffolding elements (e.g., IQGAP1) ^19,27–31^.

### Actin disorganization in INF2 disorders

Given the large excess of actin monomers (~ 100 μM) vs. regulators (0.05–4.21 μM), cells must have a tight spatio-temporal regulation of actin assembly to maintain cell polarity and shape, particularly during active proliferation and differentiation^32^. For this reason, INF2 activation must be confined to specific regions, usually at the cell cortex^6^. Here we showed that cells expressing pathogenic INF2 variants had reduced levels of actin stress fibers and cortical actin, consistent with previous reports^8,9^. Notably, the actin disorganization was morphologically more pronounced in cells expressing CMT/FSGS variants than FSGS variants, which is also consistent with the prior study^10^. The degree of cytoskeletal perturbations caused by the pathogenic INF2 variants in our fixed cell immunocytochemistry correlated with the severity of defective Ca^2+^-induced actin reorganization (Ca-AR) reported in live HeLa cells^10,26^. However, we saw no noticeable changes in filopodia in our fixed cells expressing INF2 variants, which is in contrast to prior observations indicating that constitutively active INF2 proteins increase the length of filopodia^10,14^. Our data, together with that of other investigators, indicate that CMT/FSGS variants are associated with more pronounced disorganization of actin-networks than FSGS variants. Live-cell imaging will help to clarify how *INF2* mutations affect actin-remodeling dynamics.

The diminished actin cable/bundles in HeLa cells expressing INF2 variants is consistent with some prior studies^8,9^, but conflict with other studies that showed increased actin filaments at the ER and/or cytoplasm of osteosarcoma-derived U2OS cells^12,17^. Histologic analysis of biopsied sural nerves from patients with CMT/FSGS associated with *INF2* mutations revealed that non-myelinating cells produce filopodia-like supernumerary protrusions with abnormal actin accumulation in the cytoplasm, suggesting the presence of global actinopathy. INF2 has a unique ability to promote both polymerization and depolymerization of actin^7,12,17,21,33^ (Supplementary Figure S17). Several in vitro studies with biochemical assays as well as cell transfection showed that pathogenic INF2 variants are functionally constitutively active^7,10,12,14,17,31^. However, the net effect of this constitutive activation on local actin assembly is unclear. We propose several explanations for the differences between our results and previous results. First, regulation of INF2 activity by autoinhibition in vivo may differ from that reported in vitro. Biochemical studies in vitro showed that, unlike other formins, binding of G-actin to the WH2-DAD domain relieves INF2 autoinhibition to activate actin polymerization^17,21^. In contrast, INF2 autoinhibition activity in vivo may be modified by local cellular actin monomer concentrations^29^. At lower concentrations of actin monomers than physiological levels, INF2 DID-DAD interactions inhibit polymerization, but at higher concentrations there is no inhibition^17,29^. G-actin monomer concentrations within the cell could dynamically change (1–100 μM range) in response to cofactors (e.g., actin monomer sequestering proteins like profilin and thymosin β4) capping proteins, and actin- acetylation^7,27,32^. The high affinity of INF2 WH2-DAD for G-actin allows it to serve as a sensor for subtle physiological oscillations of cytosolic G-actin levels, and to fine-tune actin polymerization in response to the cellular milieu^27^. Moreover, cellular INF2 inhibitors like CAP1 and CAP2^7,17^, as well as local demand for morphologic adaptations, may also modify actin nucleation and elongation^34^.

Second, INF2 activity would generate short and transient actin filaments^33^. In vitro*,* INF2 biochemically rapidly depolymerizes actin filaments, but whether a similar activity occurs in vivo where INF2 is abundant relative to actin, is unclear^12,17^. The severing/depolymerizing activity of INF2 could be engaged in response to the actin burst.

Third, actin disorganization caused by INF2 variants may be modified by aberrant heteromeric interactions. INF2 DID variants affect not only the actin polymerization/depolymerization equilibrium,^33^ but also crosstalk with interacting partners including members of the Rho family of small GTPases^9,19^ (Supplementary Figure S18). Inter-formin interactions are also important: mDia-DAD competes with actin monomers for INF2-DID binding *in trans*^11,29^, thereby negatively regulating mDia activity. Thus, pathogenic INF2-DID variants could diminish normal down-regulation by Rho-mDia and in turn promote mDia-mediated actin polymerization^11^.

### Role of mitochondria deficits in pathogenesis of INF2 disorders

Mitochondrial abnormalities can have significant impacts on phenotypic variability as well as severity of *INF2*-disorders. Here we found exaggerated fragmentation and peripheral misdistribution of mitochondria in cells expressing INF2 variants. Notably, these mitochondria defects were more pronounced in cells expressing CMT/FSGS variants compared to those expressing FSGS variants and the severity of mitochondrial changes correlated with the degree of cytoskeletal disorganization. Our results thus suggest that the mitochondrial defects likely reflect the dysregulated actin-microtubule network. In cells expressing INF2 variants, mitochondria tended to shift towards the cell periphery and some mitochondria aligned in parallel with disorganized microtubules. The mitochondrial misdistribution induced by INF2 variants may be due to microtubule dysfunction. Microtubules guide transport of mitochondria to specific subcellular areas such as cellular processes of neurons and podocytes^35^. Furthermore, microtubule networks have a global function that positions the Golgi complex near the centrosome and spreads the ER to the edge of cells^36^. Defective mitochondria fusion-fission at ER-mitochondria contact sites may also impair mitochondrial trafficking and tethering.

Alternatively, changes in mitochondria distribution may be associated with an intrinsic property of INF2 variants that perturbs its interactions at the ER and mitochondria. Consistent with results of our expression study, expression of the constitutively active INF2-A149D variant in HeLa cells increases mitochondria fission while reducing cell mobility and fusion events, as was seen in a prior study using U2OS cells^12,17^. Mitochondria undergo continuous fusion and fission, the balance of which determines their morphology. INF2 plays a supportive role in mitochondria fission possibly by driving initial mitochondria constriction, which facilitates Drp1-driven secondary constriction ^12^. The regulation of mitochondria dynamics also serves diverse cell functions including mitochondrial motility and trafficking, as well as interactions with other organelles such as the ER^37,38^. Up to 20% of the mitochondrial surface is in close apposition with ER membranes. This interface functions as a crucial hub for calcium signaling, apoptosis, autophagy, and lipid biosynthesis, and is thereby closely associated with cell life and death ^37^.

Podocytes and peripheral nerves have a higher energy demand relative to other cell types and thus must maintain mitochondria dynamics and distribution along their highly specialized long and/or multiple processes. Importantly, mutations in genes that are involved in mitochondrial fission (*e.g., MFN2*, *OPA1* and *GDAP1*) are a major cause of CMT^39,40^ (Supplementary Table S7). In a manner similar to *INF2*, *MFN2,* encoding a mitochondria outer-membrane protein enriched at ER-mitochondria contact sites, causes an axonal neuropathy (CMT2A2) associated with disruption of ER-mitochondria interactions and mitochondrial trafficking^39,41^. Previous histological assessment of biopsied sural nerves in patients with CMT/FSGS induced by *INF2* mutations showed no mitochondrial abnormalities, unlike those of CMT arising from *MFN2* mutations^42^. A significant fraction of axonal CMT genes are related to mitochondrial mobility, suggesting that the organelle trafficking defect is a central mechanism for CMT^38^ (Supplementary table S7).

### Conclusion

Our study with cells expressing INF2 variants suggests that mitochondrial abnormalities may be a critical consequence of actin and microtubule network disorganization. These changes together can affect the viability of both podocytes and peripheral neurons (Supplementary Figure S19). Further study to determine tissue-specific mechanisms underlying *INF2* disorders could lead to more rational therapeutic interventions for CMT/FSGS and pave the way for development of targeted and personalized medicines.

## Materials and methods

### Patients

All procedures were in accordance with the ethical standards of the committee on human study (institutional and national) and with the Helsinki Declaration. Informed consent was obtained from all subjects and/or their legal guardian(s). We enrolled 20 Japanese families and 50 sporadic cases with FSGS^43^. FSGS was defined by the presence of segmental sclerosis in some glomeruli, and the families were ascertained based on at least one individual of biopsy-proven FSGS and at least one additional blood relative affected by proteinuria (defined as proteinuria equal or higher than ++ or a urine albumin/creatinine ratio greater than 300 mg/gCr), progressive renal failure, or biopsy- proven kidney disease^1–5^. We excluded all cases of secondary FSGS because of systemic diseases (obesity, hypertension, HIV infection, and so on) or other heredity kidney diseases (Alport syndrome, thin basement membrane disease, Fabry disease, and so on).

### Mutational analysis

Genomic DNA was isolated from the peripheral blood cells using QIAamp DNA Blood isolation kit (Qiagen). We investigated in a cohort of 50 families with SRNS with or without peripheral neuropathy^43^. Index patients were screened by target panel or whole exome sequencing. Co-segregation of variants was confirmed by sequencing for family members. All rare and non-synonymous coding variants were examined. Rare variants were defined by the minor allele frequency less than 1% in the public databases. Regarding the pathogenicity of the variant, damaging effects were predicted by several algorithms including the PolyPhen2, SIFT, M-CAP, LRT, and the Mutation Taster. A systematic, tiered approach was applied for analysis and interpretation of sequence data. Called variants were interpretated and classified according to recommendation by American College of Medical Genetics and Genomics (ACMG). Variants classified as pathogenic or likely pathogenic (category 1. or 2. according to ACMG) were further analyzed.

### Constructs and mutagenesis

The cDNA constructs, in which the human full length *INF2* (NM_022489, the prenylated, CAAX isoform) was subcloned into the pcDNA 3.1 vectors with either cMyc or GFP tags, were used. For immunocytochemistry and western analysis, N-terminal cMyc-tagged *INF2* (GeneCopoeia Ex-Z4823- M43) was used as the wild-type. The single base substitutions of INF2 variants G73D, V108D, T161N, R218W, and N202S were introduced by artificial DNA synthesis for fragments flanked by SalI or Hind III sites (Genescript). Mutagenesis reactions were verified for both strands by Sanger sequencing. For live cell imaging, the ORF clone of pcDNA3.1 + N-eGFP (Genescript ID: OHu18878C) was used as the wild-type. Single nucleotide substitutions p.G73D and A p.T161N substitution was introduced by artificial DNA synthesis of KpnI/XhoI sites and enzyme sites, respectively. Vector containing scrambled oligonucleotides (OmicsLink Expression Clone: EX-NEG-M43) was used as transfection control.

### Structural and molecular dynamics analysis

#### 3D structure modeling

All the variants we identified lie within the DID, which comprises an autoinhibitory domain in the diaphanous formin. In this context, we utilized the structures of the self-autoinhibited mDia1 DID/DAD complex (PDB:2F31), in which the N-terminal DID interacts with the C-terminal DAD, thereby serving the autoinhibition of mDia functions^16^. Three dimensional structure of the human *INF2* was modelled based on the crystal structure of the mouse mDia PDB 2F31. The modelling was performed by the comparative modeling method using the Rosetta program on the Robetta platform (http://new.robetta.org). To assess the potential structural effects of *INF2* mutations, we modeled the INF2 variants based on wild-type model using myPresto function with 10,000 rounds of minimization. Interface area (Å) and free energy (ΔG) of pathogenic INF2 variants were predicted using PISA software (https://www.ebi.ac.uk/pdbe/pisa/)^44^. Protein–Protein interaction diagrams were predicted by the PDBsum program^45^. For the comparison of our modeled structure and AlphaFold one, the model of human INF2 was retrieved from AlphaFold protein structure database (https://alphafold.ebi.ac.uk).

#### Molecular dynamics simulations

MD simulations of the wild-type INF2 and variants were performed under periodic boundary conditions using GROMACS 2022.5, Amber ff99SB force field for proteins and ions, and TIP3P model for water molecules ^46,47^. Water molecules were placed around the protein model in a 2 nm box size containing approximately 26,000 water molecules. Ions were placed at 150 mM, the same as in vivo, and SLTCAP was used to calculate the number of ions^48,49^.

Electrostatic interactions were calculated using the particle mesh Ewald (PME) method with a cutoff radius of 1.0 nm. Van der Waals interactions were also cut off at 1.0 nm. The LINKS algorithm was used for bond length fixation. The Steepest Descent method was used for energy minimization, the V-rescale method was used to control the equilibration temperature to 310 K, and the Parrinello-Rahman method was used to maintain the pressure at 105 Pa. First, the energy of the system was minimized to 1000.0 kJ/mol/nm by energy minimization, followed by 200 ps of NVT equilibrium and 800 ps of NPT equilibrium, and then a 500 ns production run was performed. The trajectory output was extracted every 1 ns, where the time-step of 2 fs was set for the all simulations (500,000 steps). We compared the molecular dynamics between wild-type INF2 and pathogenic variants by capturing and aligning the images with VMD software (http://www.ks.uiuc.edu/Research/vmd/). Tracing for the dynamic changes in positions of residues in the DID and DAD interface during 500 ns (250,000,000 steps). For the RMSF analysis, the results after 100 ns, when the RMSD of the MD calculation stabilized were used^50^.

### Cellular phenotype imaging and analysis

#### Cell lines

HeLa cells (RCB0007, RIKEN BRC) or COS-7 (RCB0539) were grown in DMEM medium (Wako, 043-30085) with 10% fetal bovine serum. The cells were maintained under normal culture conditions with 5% CO2 and 37 °C of temperature and medium was changed every 48 h until reached 80% confluency. At this point the cells were gently washed with PBS (−) and trypsinization using 1 ml TrypLE Express (Gibco, 12605-101).

#### Immunofluorescence staining for actin filaments

Cells grown on coverslips (1 × 10^5^ cells in 12 well plate) were transfected with 1 μg of plasmids constructs, either wild-type INF2 or pathogenic variants, by use of TransIT-LT1 (Mirus, MIR 2300). After 8 h transfection, cells were fixed in 4% PFA in PBS for 20 min at room temperature. After rinsing in PBS, cells were permeabilized with 0.1% Triton X-100 for 10 min, followed by blocking in 5% goat serum (Wako, 143-06561) for 1 h at room temperature. cMyc-tagged INF2 was visualized by primary monoclonal anti-cMyc (1:1,000, Sigma, #4439) antibody and fluorescent- labeled secondary antibody (1:1000, anti-mouse Alexa 488) for 60 min at room temperature. For actin filaments detection, phalloidin-conjugated fluorescent 633 (1:50, Thermofisher, A22284) was added to the secondary antibody solution. Nuclei were counterstained using DAPI and coverslips were mounted with Prolong Gold antifade reagent (Invitrogen, P36930). The immunofluorescence images were obtained by confocal microscope Olympus FV3000 and focus on actin filaments at the peripheral area. The photographs were captured by using a 63X/1.4 Plan Apo Oil matched with the large-format 1024 × 1024 pixel.

Staining pattern of stress fibers by phalloidin were classified into the four subgroups based upon the morphologic features : types A (> 90% of cell area filled with thick cables), type B (at least 2 thick cables running under nucleus and rest of cell area filled with fine cables) staining patterns, type C (no thick cables, but some cables present) and type D (no cables visible in the central area of the cell)^22,23^. Images were analyzed by visual inspection or automated classification by us of machine learning.

Approximately 500 cells were counted per transfection construct for in 3–6 independent experiments.

#### Immunofluorescence staining for mitochondria

For mitochondrial labeling in live-imaging, cells were seeded in a glass-bottom dish (35 mm) with the cell density of 3 × 10^5^ cells for 24 h to reached 80% confluency and the medium was changed before transfection. The cells were transfected with 2.5 μg of GFP-tagged INF2 plasmids onto the cells by using Lipofectamine P3000 and incubated at 37 °C for 8 h. After that, the cells were labeled with MitoTracker Red CMXRos (Thermofisher, M7512) at 100 nM concentration for 15 min at room temperature in phenol-red-free DMEM. Live cell imaging was done by confocal microscopy Zeiss LSM700. To visualize the dynamic of mitochondria, time-lapse images were obtained with every 5 s interval for 10 min.

To classify the mitochondria pattern, cells were fixed with 4% paraformaldehyde. The morphologic patterns were quantified by classifying the appearance of mitochondria into three subclasses (tubular, mixed, and fragmented) in transfected cells according to the protocol previously described^51,52^. To analyze the 2D mitochondria motility, we took a representative stack of the cell (20 cells/each type) and measured mitochondrial length, maximum intensity projections of z-series with 0.5 μm increments for red channel (MitoTracker)^12^. The flat regions of cells with clearly resolved mitochondria were selected, and 10 mitochondria per cell were measured using the line tool in NIH image software. For intensity profiling, the intensity profiles of mitochondrial signals along the lines were plotted by ImageJ. Images were digitally converted to grayscale 8-bit and analyzed by use of the surface plot command. A 3D graph was reconstituted as Y axis representing the intensity (gray value) of the mitochondria, whereas X-axis and Z-axis demonstrated the cell shape and size, respectively.

#### Transfer learning on deep convolutional neural network (CNN)

We initialized our network parameters to default parameter set provided for the deep CNN, ALEXNET, and then fine-tuned the parameters of the last layer of the networks on our data via back propagation by using two NVIDIA GeForce RTX 2080Ti graphic processing units. The loss function was defined as the cross entropy between predicted probability and the true class labels, and we used SGDM optimization with learning rate of 0.0001 and momentum of 0.9 for training the weights.

For the mitochondria morphology classification, we obtained 1,024 × 1,024 pixel of 34 images for INF2 wild-type and p.T161N variant, respectively. After clipping the transfected HeLa cell images according to the GFP signals, we randomly extracted 227 × 227 pixel of 6,800 (100 × 34 × 2) images from the original images as the ALEXNET input and used 7% (400/6,800) of those for training.

For the actin morphology classification, we obtained 56 wild-type and 79 mutant expressing cell images and used 3% (400) of the extracted 227 × 227 pixel of 13,500 (100 × (56 + 79)) images for training. Statistical differences in morphology scores between the wild-type and p.T161N variants were evaluated with Wilcoxon rank-sum test.

#### Immunofluorescence staining for microtubule and phenotyping classification

To stain the microtubule, cells were firstly labeled with anti-tubulin antibody (1:300, Abcam, ab7291) and further incubated with the fluorescence labeled secondary antibody (1:1000, Abcam, ab150115). By visual inspection of immunofluorescence staining of β-tubulin, subcellular patterns of were classified into 3 subgroups by use of the following criteria: (i) oriented in a radiating array extended from centrosome area near the nuclei (the microtubule organizing center) to the periphery (normal subtype), (ii) intermediate, heterogeneous mixed configuration consists of normal radiating and thick bundles disarranged in parallel (intermediate subtype), and (iii) most aligned along the long axis of the cells (disorganized subtype)^53^. All images were scored by at least three independent scorers.

### Statistics

Data were expressed as mean ± SEM. Statistical analysis was performed using 2-tailed Student’s t test or one-way ANOVA (SigmaStat, version 3.1.1). Survival analysis was carried out using the log- rank test (Prism 8; GraphPad). The pairwise differences in a proportional distribution of four actin-phenotypic categories between wild-type INF2 and variants were analyzed by use of Fisher exact test on the R program with multiple testing corrections. *P* < 0.05 was considered significant.

### Western Analysis

Cells were homogenized by Dounce homogenizer in RIPA buffer (50 mM Tris, pH 7.4, 150 mM NaCl, 0.1% SDS, 0.5% Na deoxycholate, 1% Triton X-100, and protease inhibitors) supplemented with SIGMAFAST protease inhibitor Tablets (Millipore-Sigma). Lysates were cleaned by centrifugation at 13,000 g, for 20 min. After heating at 95 °C 5 min in Laemmli sample buffer containing β- mercapto-ethanol, approximately 25 µg samples per well were loaded and separated by 7.5% SDS- PAGE gels. The proteins were then transferred to the polyvinylidene difluoride membrane (PVDF) membranes by Mini Trans-Blot Cell (Bio-Rad) (30 V, 6 h). The membrane was blocked with 5% BSA for 1 h, then incubated with primary antibodies (anti-cMyc 9106, 1:100, or anti-β tubulin, 1:300) at 4 °C overnight. After washing with PBS-T, the membrane was then incubated with horseradish peroxidase (HRP)-conjugated secondary antibodies (1:5,000) (NA931, Amersham) for 1 h at room temperature. Signals were detected by chemo-luminescence and were captured using an imaging system (LAS-4000, Fujifilm).

### Clinical details of the five index cases with CMT/FSGS

#### Family ID 7-OK

*Patient (II-1)* first noticed proteinuria at age 11 on regular check-up and exhibited SRNS at age 11 year^54^. Renal biopsy revealed FSGS with the tubule-interstitial injuries. Despite the immunosuppressive therapy, she eventually progressed into ESRD at age 14. She first noticed difficulty in walking around at age 14. Neurological assessment at age of 15 revealed that she had steppage gait with *pes cavus* deformity and distal muscle weakness. Muscle atrophy was noted in the upper and lower extremities, and deep tendon reflexes was decreased in the lower extremities. No sensory disturbance was noted.

*Patient (II-3)*, a younger brother of case II-1. No parental consanguinity was noted. He first manifested proteinuria at age 6. The proteinuria gradually increased to nephrotic level at age 11. He underwent renal biopsy at age 11 with a diagnosis of FSGS and eventually progressed to ESRD at age 15. Neurological assessment at age 12 demonstrated distal muscle weakness with steppage gait, and muscle atrophy in the upper and lower extremities. Deep tendon reflexes were decreased in the lower extremities. There were no sensory disturbances in peripheral neurons.

#### Family ID 8 NI

*Patient (II-1)* first noticed proteinuria at age 11 and developed nephrotic syndrome at age 14^55^. Renal biopsy at age 14 revealed FSGS. The renal disease was resistant to steroid therapy and progressed into ESRD at age 16. She had difficulty in walking since childhood. *Pes cavus* deformity was corrected by orthopedic surgery at age 11. Neurological examination at age 14 demonstrated the steppage gait with asymmetric atrophy and weakness in the peroneal and tibial muscles. An audiogram revealed a bilateral sensorineural deafness with hearing loss of 50 dB. Various auditory testings suggested that the hearing inability is ascribed to the auditory periphery, cochlea, or auditory nerves.

#### Family ID 9 OS

*Patient (II-1)* first manifested proteinuria on regular check-up at age 11. He developed nephrotic proteinuria at age 13 with deteriorating renal functions. Renal biopsy at age 14 revealed FSGS in the absence of immune-depositions. He had been treated with steroids and angiotensin converting enzyme inhibitors and angiotensin receptor blockers in combination. The renal disease was resistant to the steroids with progressive functional deterioration and eventually reached ESRD at age 14. He noticed difficulty in walking at age 15. On neurological examination at age 15, he exhibited muscle weakness and atrophy predominantly in the lower extremities and had steppage gait with drop foot, and *pes cavus* deformity. INF2 variants was reported in mutational survey of SRNS^56^.

#### Family ID 10 YA

*Patient (II-1)* first presented proteinuria at regular check-up at age 14^54^. The first renal biopsy was diagnosed as MCNS. Because of deteriorating renal function and increased proteinuria, he underwent the second renal biopsy at age 16, revealing a FSGS histology. The renal disease was resistant to steroid therapy and progressed into ESRD at age 17. He first noticed difficulty in walking at age 10 and was diagnosed as CMT. On neurological examination at age 17, muscle atrophy was noted in the distal muscles of the forearm and lower limb with absent distal deep tendon reflexes. He had progressive gait disturbance with steppage gait. Neither intellectual nor hearing disability were noted. Biopsy of peroneal nerve revealed degeneration in large myelinated fibers with onion bulb formation, which is typically seen for demyelinating CMT1 subtype.

### Ethical approval

The research related to human subject has been complied with all the relevant national regulations, institutional policies, and in accordance with the Helsinki Declaration, and has been approved by the institutional review board in the Kansai Medical University (2008902) and each affiliation.

## Supplementary Information

Supplementary Tables.

Supplementary Information 2.

## Data Availability

The datasets generated and/or analyzed during the current study are available in the temporary review repository [Ueda & Quynh Submission Template], at https://drive.google.com/drive/folders/1lvFWS6hiOVqPHuv-y8Sat-FiIMXSy3X_?usp=sharing.
