# Supplementary material for: Characterization of cytoskeletal and structural effects of INF2 variants causing glomerulopathy and neuropathy
Source: Sci Rep. 2023 Jul 25;13:12003. doi: 10.1038/s41598-023-38588-7 (PMC10368640; doi:10.1038/s41598-023-38588-7)
Supplement: Supplementary file 1 — Supplementary Tables. [file 41598_2023_38588_MOESM1_ESM.pptx]

## Slide 1
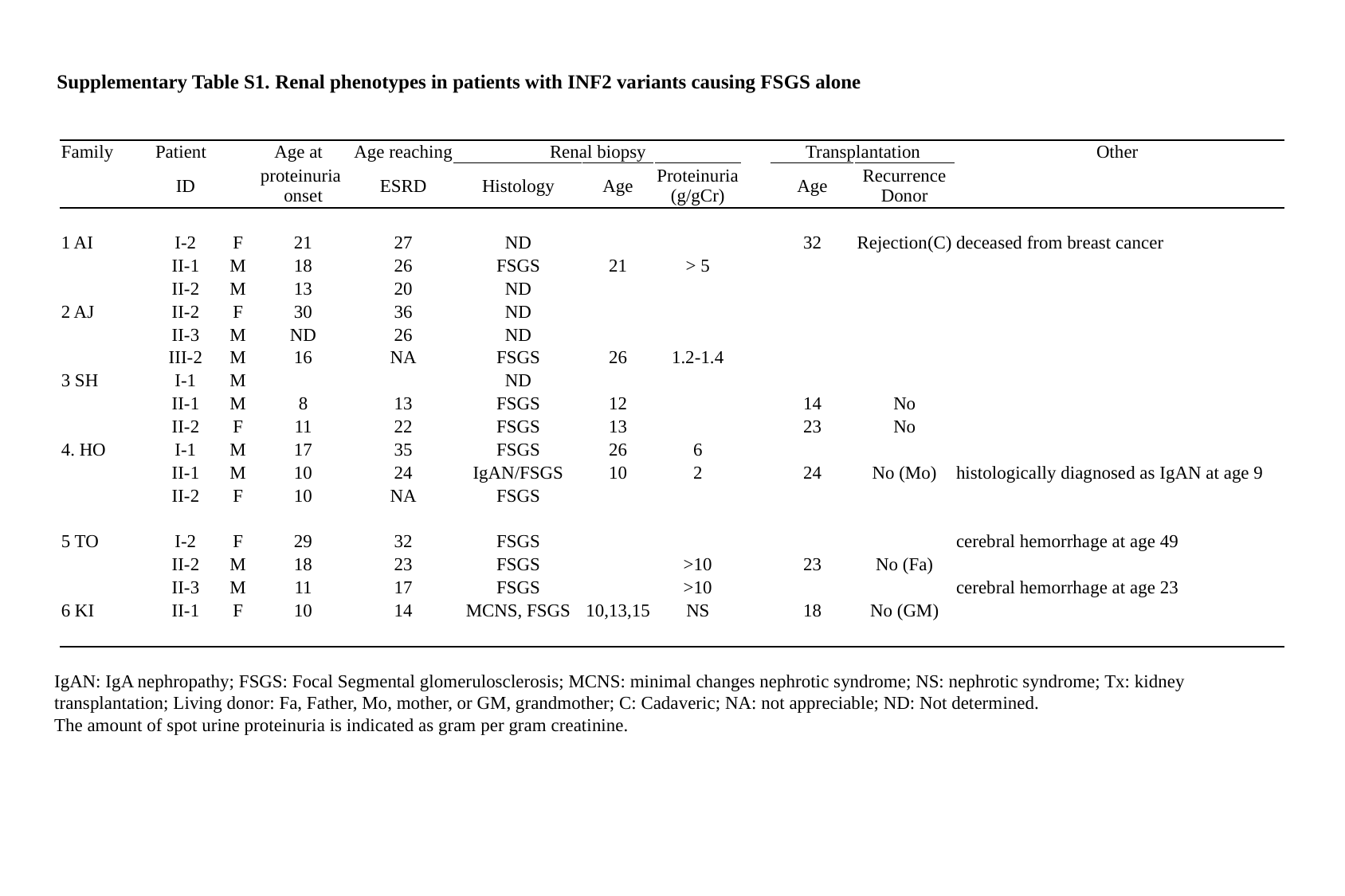

Supplementary Table S1. Renal phenotypes in patients with INF2 variants causing FSGS alone
| | | | | | | | | | | | | | | |
| --- | --- | --- | --- | --- | --- | --- | --- | --- | --- | --- | --- | --- | --- | --- |
| Family | Patient | | Age at | Age reaching | Renal biopsy | | | | | | Transplantation | | | Other |
| | ID | | proteinuria onset | ESRD | Histology | Age | age | Proteinuria (g/gCr) | proteinuria (g/gCr) | | Age | Recurrence Donor | Recurrence Donor | |
| | | | | | | | | | | | | | | |
| 1 AI | I-2 | F | 21 | 27 | ND | | | | | | 32 | Rejection(C) | | deceased from breast cancer |
| | II-1 | M | 18 | 26 | FSGS | 21 | | > 5 | | | | | | |
| | II-2 | M | 13 | 20 | ND | | | | | | | | | |
| 2 AJ | II-2 | F | 30 | 36 | ND | | | | | | | | | |
| | II-3 | M | ND | 26 | ND | | | | | | | | | |
| | III-2 | M | 16 | NA | FSGS | 26 | | 1.2-1.4 | 1.2-1.4 | | | | | |
| 3 SH | I-1 | M | | | ND | | | | | | | | | |
| | II-1 | M | 8 | 13 | FSGS | 12 | | | | | 14 | No | No | |
| | II-2 | F | 11 | 22 | FSGS | 13 | | | | | 23 | No | No | |
| 4. HO | I-1 | M | 17 | 35 | FSGS | 26 | | 6 | 6 | | | | | |
| | II-1 | M | 10 | 24 | IgAN/FSGS | 10 | | 2 | 2 | | 24 | No (Mo) | No (Mo) | histologically diagnosed as IgAN at age 9 |
| | II-2 | F | 10 | NA | FSGS | | | | | | | | | |
| | | | | | | | | | | | | | | |
| 5 TO | I-2 | F | 29 | 32 | FSGS | | | | | | | | | cerebral hemorrhage at age 49 |
| | II-2 | M | 18 | 23 | FSGS | | | >10 | | | 23 | No (Fa) | No (Fa) | |
| | II-3 | M | 11 | 17 | FSGS | | | >10 | | | | | | cerebral hemorrhage at age 23 |
| 6 KI | II-1 | F | 10 | 14 | MCNS, FSGS | 10,13,15 | | NS | | | 18 | No (GM) | | |
| | | | | | | | | | | | | | | |
IgAN: IgA nephropathy; FSGS: Focal Segmental glomerulosclerosis; MCNS: minimal changes nephrotic syndrome; NS: nephrotic syndrome; Tx: kidney transplantation; Living donor: Fa, Father, Mo, mother, or GM, grandmother; C: Cadaveric; NA: not appreciable; ND: Not determined.
The amount of spot urine proteinuria is indicated as gram per gram creatinine.

## Slide 2
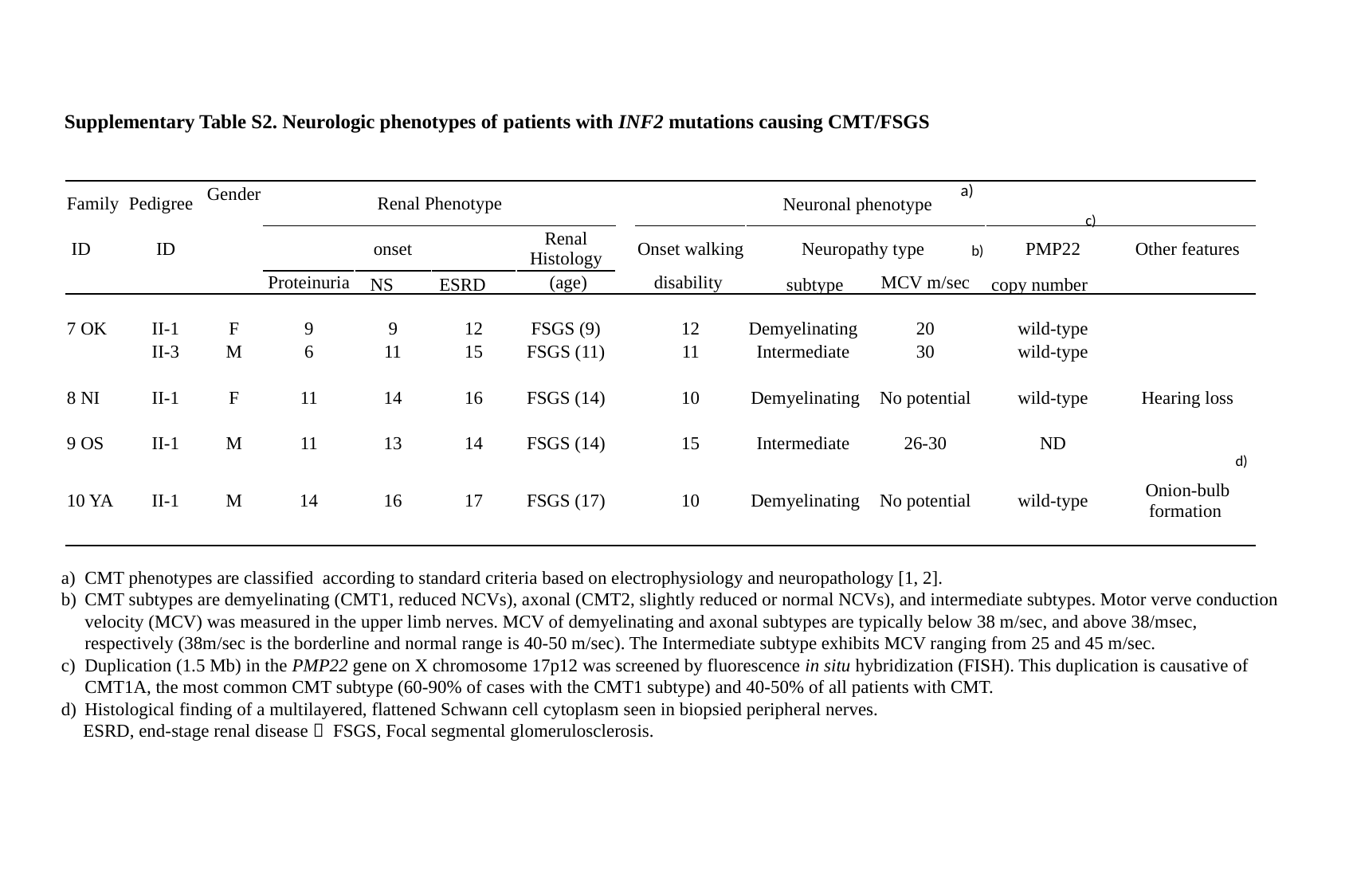

Supplementary Table S2. Neurologic phenotypes of patients with INF2 mutations causing CMT/FSGS
| | | | | | | | | | | | | |
| --- | --- | --- | --- | --- | --- | --- | --- | --- | --- | --- | --- | --- |
| Family | Pedigree | Gender | Renal Phenotype | | | | | Neuronal phenotype | | | | |
| ID | ID | | | onset | | Renal Histology | | Onset walking | Neuropathy type | | PMP22 | Other features |
| | | | Proteinuria | NS | ESRD | (age) | | disability | subtype | MCV m/sec | copy number | |
| | | | | | | | | | | | | |
| 7 OK | II-1 | F | 9 | 9 | 12 | FSGS (9) | | 12 | Demyelinating | 20 | wild-type | |
| | II-3 | M | 6 | 11 | 15 | FSGS (11) | | 11 | Intermediate | 30 | wild-type | |
| | | | | | | | | | | | | |
| 8 NI | II-1 | F | 11 | 14 | 16 | FSGS (14) | | 10 | Demyelinating | No potential | wild-type | Hearing loss |
| | | | | | | | | | | | | |
| 9 OS | II-1 | M | 11 | 13 | 14 | FSGS (14) | | 15 | Intermediate | 26-30 | ND | |
| | | | | | | | | | | | | |
| 10 YA | II-1 | M | 14 | 16 | 17 | FSGS (17) | | 10 | Demyelinating | No potential | wild-type | Onion-bulb formation |
| | | | | | | | | | | | | |
| | | | | | | | | | | | | |
a)
c)
b)
d)
CMT phenotypes are classified according to standard criteria based on electrophysiology and neuropathology [1, 2].
CMT subtypes are demyelinating (CMT1, reduced NCVs), axonal (CMT2, slightly reduced or normal NCVs), and intermediate subtypes. Motor verve conduction velocity (MCV) was measured in the upper limb nerves. MCV of demyelinating and axonal subtypes are typically below 38 m/sec, and above 38/msec, respectively (38m/sec is the borderline and normal range is 40-50 m/sec). The Intermediate subtype exhibits MCV ranging from 25 and 45 m/sec.
Duplication (1.5 Mb) in the PMP22 gene on X chromosome 17p12 was screened by fluorescence in situ hybridization (FISH). This duplication is causative of CMT1A, the most common CMT subtype (60-90% of cases with the CMT1 subtype) and 40-50% of all patients with CMT.
Histological finding of a multilayered, flattened Schwann cell cytoplasm seen in biopsied peripheral nerves.
	ESRD, end-stage renal disease； FSGS, Focal segmental glomerulosclerosis.

## Slide 3
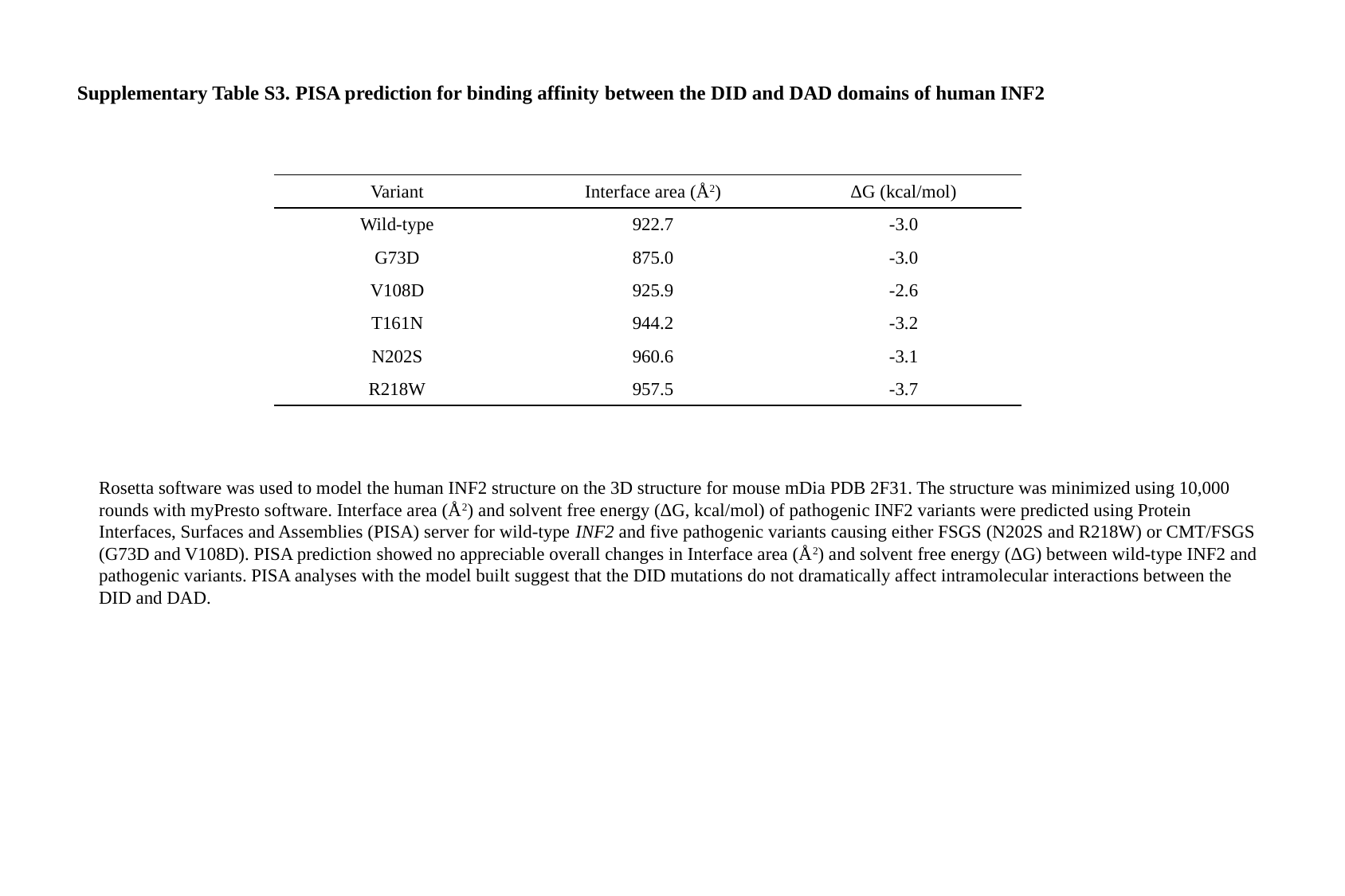

Supplementary Table S3. PISA prediction for binding affinity between the DID and DAD domains of human INF2
| | | |
| --- | --- | --- |
| Variant | Interface area (Å2) | ΔG (kcal/mol) |
| Wild-type | 922.7 | -3.0 |
| G73D | 875.0 | -3.0 |
| V108D | 925.9 | -2.6 |
| T161N | 944.2 | -3.2 |
| N202S | 960.6 | -3.1 |
| R218W | 957.5 | -3.7 |
| | | |
Rosetta software was used to model the human INF2 structure on the 3D structure for mouse mDia PDB 2F31. The structure was minimized using 10,000 rounds with myPresto software. Interface area (Å2) and solvent free energy (ΔG, kcal/mol) of pathogenic INF2 variants were predicted using Protein Interfaces, Surfaces and Assemblies (PISA) server for wild-type INF2 and five pathogenic variants causing either FSGS (N202S and R218W) or CMT/FSGS (G73D and V108D). PISA prediction showed no appreciable overall changes in Interface area (Å2) and solvent free energy (ΔG) between wild-type INF2 and pathogenic variants. PISA analyses with the model built suggest that the DID mutations do not dramatically affect intramolecular interactions between the DID and DAD.

## Slide 4
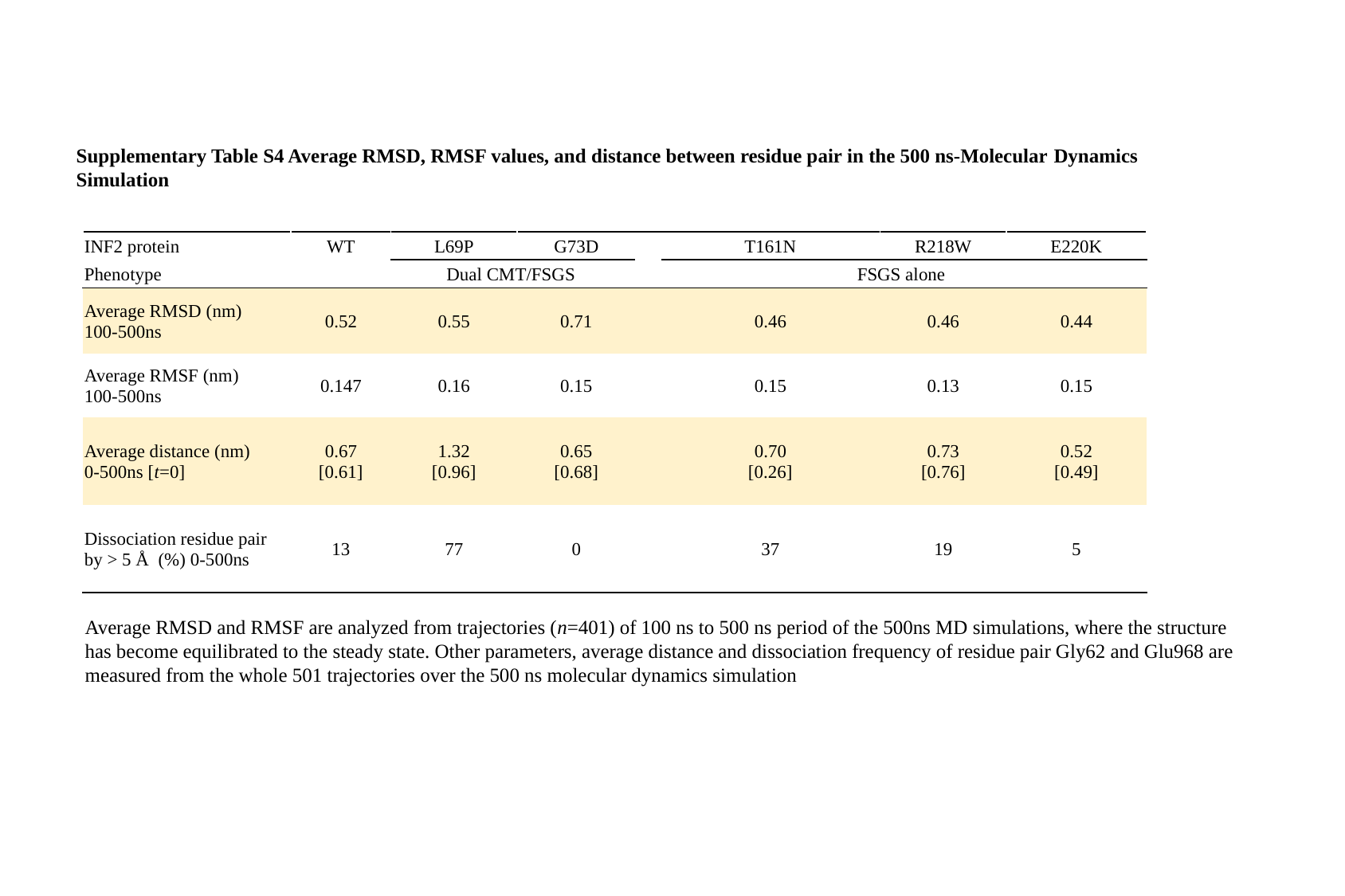

Supplementary Table S4 Average RMSD, RMSF values, and distance between residue pair in the 500 ns-Molecular Dynamics Simulation
| INF2 protein | WT | L69P | G73D | | T161N | R218W | E220K |
| --- | --- | --- | --- | --- | --- | --- | --- |
| Phenotype | | Dual CMT/FSGS | | | FSGS alone | | |
| Average RMSD (nm) 100-500ns | 0.52 | 0.55 | 0.71 | | 0.46 | 0.46 | 0.44 |
| Average RMSF (nm) 100-500ns | 0.147 | 0.16 | 0.15 | | 0.15 | 0.13 | 0.15 |
| Average distance (nm) 0-500ns [t=0] | 0.67 [0.61] | 1.32 [0.96] | 0.65 [0.68] | | 0.70 [0.26] | 0.73 [0.76] | 0.52 [0.49] |
| Dissociation residue pair by > 5 Å (%) 0-500ns | 13 | 77 | 0 | | 37 | 19 | 5 |
| | | | | | | | |
Average RMSD and RMSF are analyzed from trajectories (n=401) of 100 ns to 500 ns period of the 500ns MD simulations, where the structure has become equilibrated to the steady state. Other parameters, average distance and dissociation frequency of residue pair Gly62 and Glu968 are measured from the whole 501 trajectories over the 500 ns molecular dynamics simulation

## Slide 5
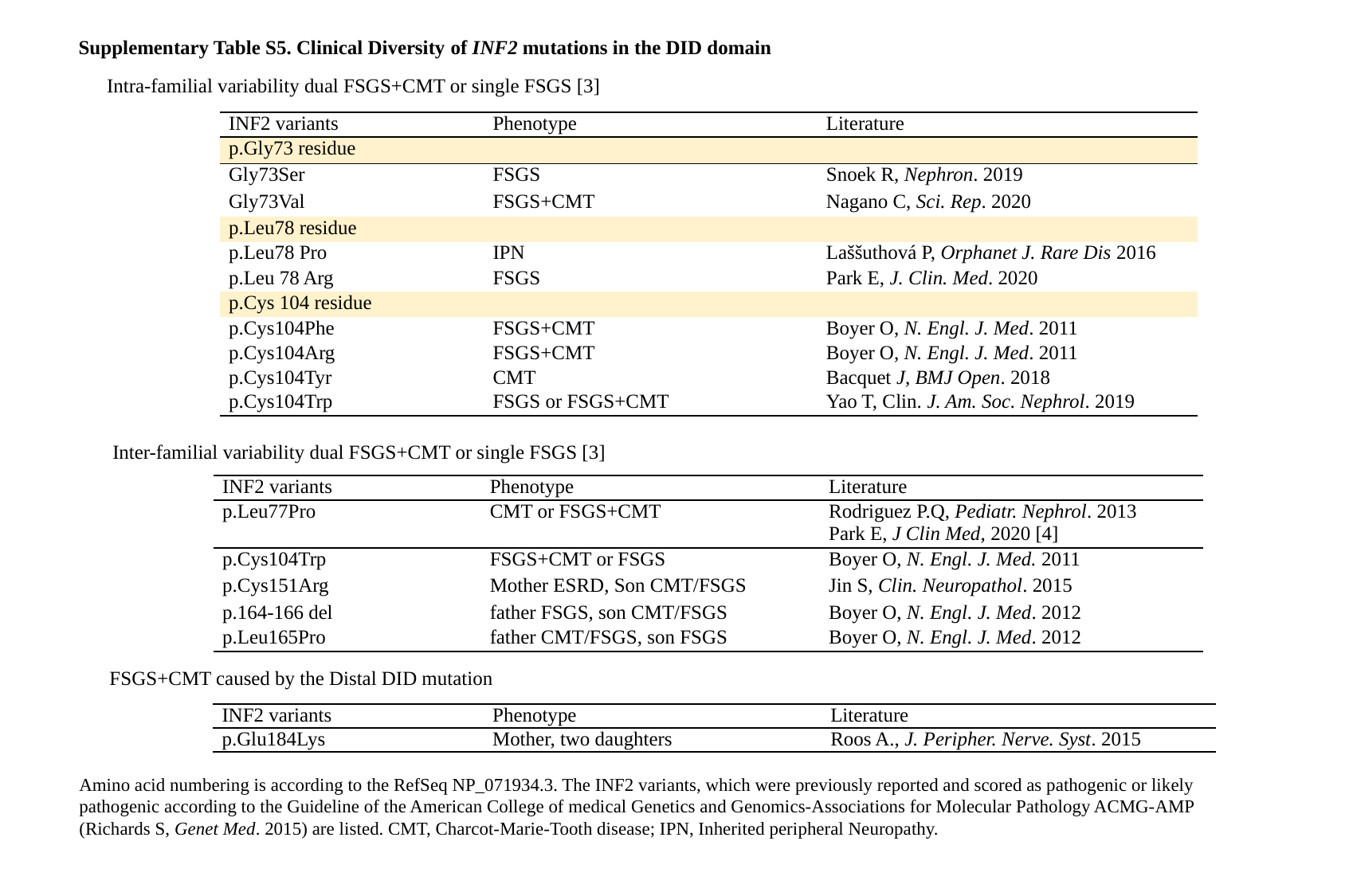

Supplementary Table S5. Clinical Diversity of INF2 mutations in the DID domain
Intra-familial variability dual FSGS+CMT or single FSGS [3]
| INF2 variants | Phenotype | Literature |
| --- | --- | --- |
| p.Gly73 residue | | |
| Gly73Ser | FSGS | Snoek R, Nephron. 2019 |
| Gly73Val | FSGS+CMT | Nagano C, Sci. Rep. 2020 |
| p.Leu78 residue | | |
| p.Leu78 Pro | IPN | Laššuthová P, Orphanet J. Rare Dis 2016 |
| p.Leu 78 Arg | FSGS | Park E, J. Clin. Med. 2020 |
| p.Cys 104 residue | | |
| p.Cys104Phe | FSGS+CMT | Boyer O, N. Engl. J. Med. 2011 |
| p.Cys104Arg | FSGS+CMT | Boyer O, N. Engl. J. Med. 2011 |
| p.Cys104Tyr | CMT | Bacquet J, BMJ Open. 2018 |
| p.Cys104Trp | FSGS or FSGS+CMT | Yao T, Clin. J. Am. Soc. Nephrol. 2019 |
Inter-familial variability dual FSGS+CMT or single FSGS [3]
| INF2 variants | Phenotype | Literature |
| --- | --- | --- |
| p.Leu77Pro | CMT or FSGS+CMT | Rodriguez P.Q, Pediatr. Nephrol. 2013 Park E, J Clin Med, 2020 [4] |
| p.Cys104Trp | FSGS+CMT or FSGS | Boyer O, N. Engl. J. Med. 2011 |
| p.Cys151Arg | Mother ESRD, Son CMT/FSGS | Jin S, Clin. Neuropathol. 2015 |
| p.164-166 del | father FSGS, son CMT/FSGS | Boyer O, N. Engl. J. Med. 2012 |
| p.Leu165Pro | father CMT/FSGS, son FSGS | Boyer O, N. Engl. J. Med. 2012 |
FSGS+CMT caused by the Distal DID mutation
| INF2 variants | Phenotype | Literature |
| --- | --- | --- |
| p.Glu184Lys | Mother, two daughters | Roos A., J. Peripher. Nerve. Syst. 2015 |
Amino acid numbering is according to the RefSeq NP_071934.3. The INF2 variants, which were previously reported and scored as pathogenic or likely pathogenic according to the Guideline of the American College of medical Genetics and Genomics-Associations for Molecular Pathology ACMG-AMP (Richards S, Genet Med. 2015) are listed. CMT, Charcot-Marie-Tooth disease; IPN, Inherited peripheral Neuropathy.

## Slide 6
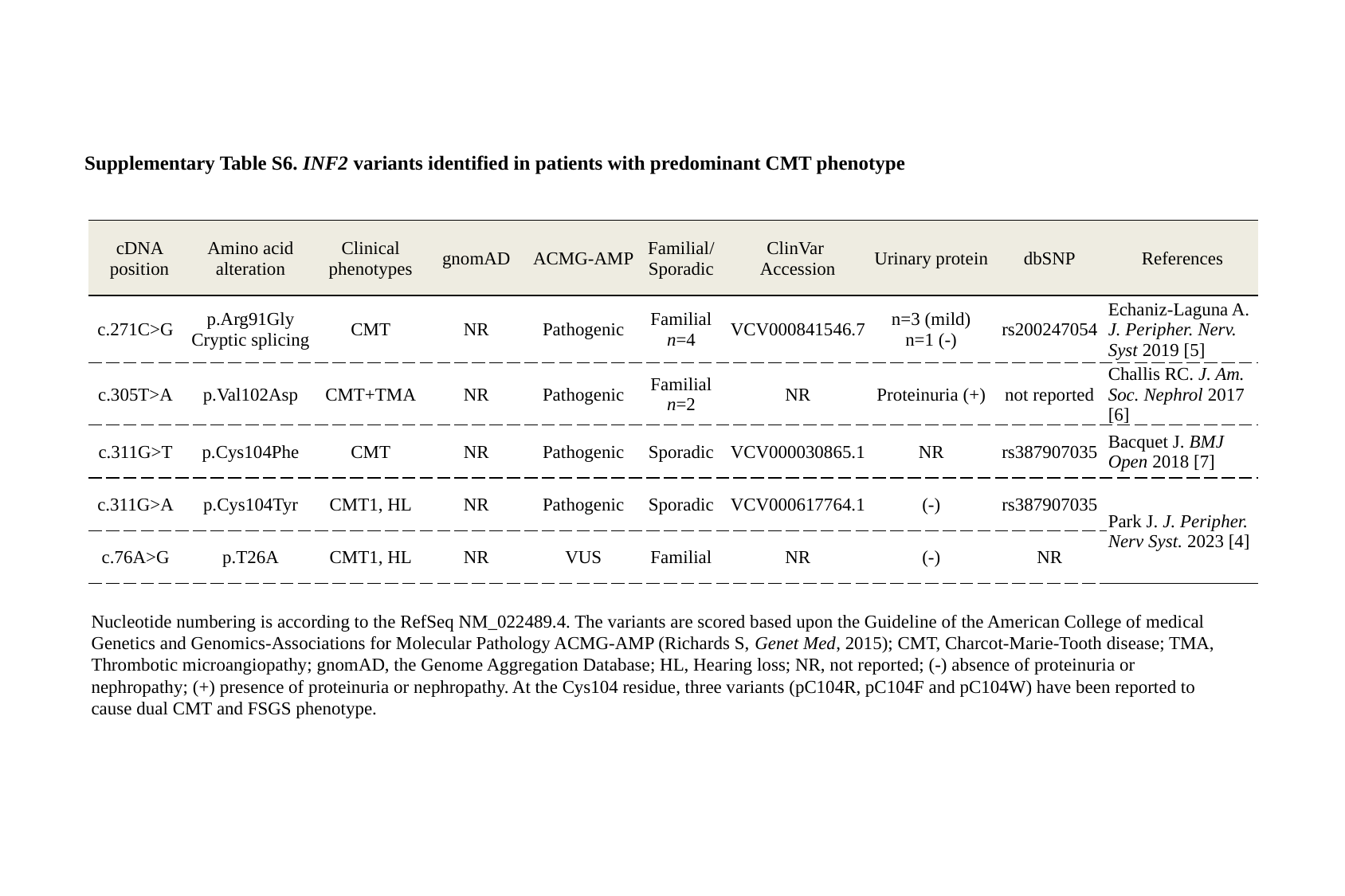

Supplementary Table S6. INF2 variants identified in patients with predominant CMT phenotype
| cDNA position | Amino acid alteration | Clinical phenotypes | gnomAD | ACMG-AMP | Familial/ Sporadic | ClinVar Accession | Urinary protein | dbSNP | References |
| --- | --- | --- | --- | --- | --- | --- | --- | --- | --- |
| c.271C>G | p.Arg91Gly Cryptic splicing | CMT | NR | Pathogenic | Familial n=4 | VCV000841546.7 | n=3 (mild) n=1 (-) | rs200247054 | Echaniz-Laguna A. J. Peripher. Nerv. Syst 2019 [5] |
| c.305T>A | p.Val102Asp | CMT+TMA | NR | Pathogenic | Familial n=2 | NR | Proteinuria (+) | not reported | Challis RC. J. Am. Soc. Nephrol 2017 [6] |
| c.311G>T | p.Cys104Phe | CMT | NR | Pathogenic | Sporadic | VCV000030865.1 | NR | rs387907035 | Bacquet J. BMJ Open 2018 [7] |
| c.311G>A | p.Cys104Tyr | CMT1, HL | NR | Pathogenic | Sporadic | VCV000617764.1 | (-) | rs387907035 | Park J. J. Peripher. Nerv Syst. 2023 [4] |
| c.76A>G | p.T26A | CMT1, HL | NR | VUS | Familial | NR | (-) | NR | |
Nucleotide numbering is according to the RefSeq NM_022489.4. The variants are scored based upon the Guideline of the American College of medical Genetics and Genomics-Associations for Molecular Pathology ACMG-AMP (Richards S, Genet Med, 2015); CMT, Charcot-Marie-Tooth disease; TMA, Thrombotic microangiopathy; gnomAD, the Genome Aggregation Database; HL, Hearing loss; NR, not reported; (-) absence of proteinuria or nephropathy; (+) presence of proteinuria or nephropathy. At the Cys104 residue, three variants (pC104R, pC104F and pC104W) have been reported to cause dual CMT and FSGS phenotype.

## Slide 7
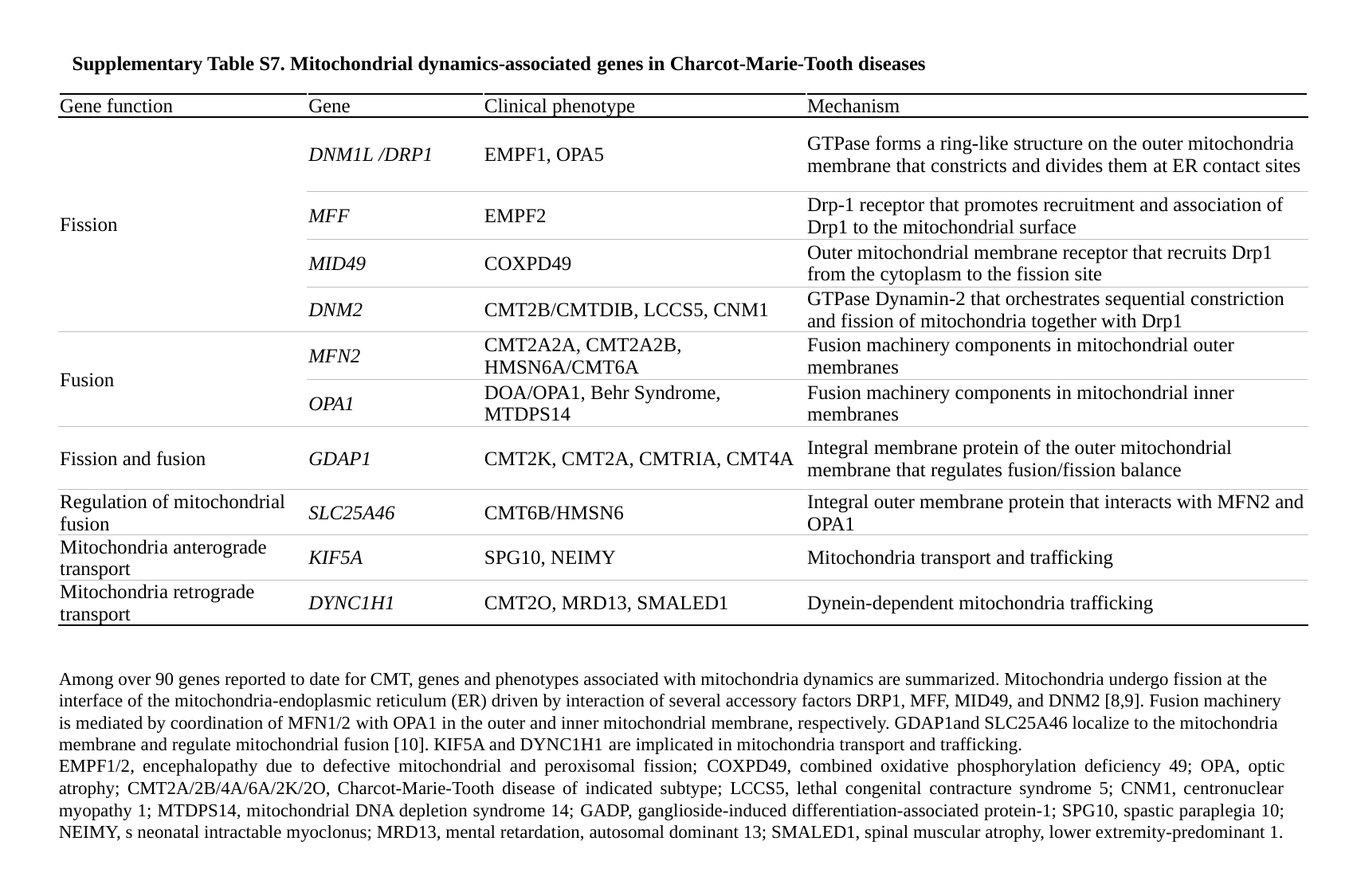

Supplementary Table S7. Mitochondrial dynamics-associated genes in Charcot-Marie-Tooth diseases
| Gene function | Gene | Clinical phenotype | Mechanism |
| --- | --- | --- | --- |
| Fission | DNM1L /DRP1 | EMPF1, OPA5 | GTPase forms a ring-like structure on the outer mitochondria membrane that constricts and divides them at ER contact sites |
| | MFF | EMPF2 | Drp-1 receptor that promotes recruitment and association of Drp1 to the mitochondrial surface |
| | MID49 | COXPD49 | Outer mitochondrial membrane receptor that recruits Drp1 from the cytoplasm to the fission site |
| | DNM2 | CMT2B/CMTDIB, LCCS5, CNM1 | GTPase Dynamin-2 that orchestrates sequential constriction and fission of mitochondria together with Drp1 |
| Fusion | MFN2 | CMT2A2A, CMT2A2B, HMSN6A/CMT6A | Fusion machinery components in mitochondrial outer membranes |
| | OPA1 | DOA/OPA1, Behr Syndrome, MTDPS14 | Fusion machinery components in mitochondrial inner membranes |
| Fission and fusion | GDAP1 | CMT2K, CMT2A, CMTRIA, CMT4A | Integral membrane protein of the outer mitochondrial membrane that regulates fusion/fission balance |
| Regulation of mitochondrial fusion | SLC25A46 | CMT6B/HMSN6 | Integral outer membrane protein that interacts with MFN2 and OPA1 |
| Mitochondria anterograde transport | KIF5A | SPG10, NEIMY | Mitochondria transport and trafficking |
| Mitochondria retrograde transport | DYNC1H1 | CMT2O, MRD13, SMALED1 | Dynein-dependent mitochondria trafficking |
Among over 90 genes reported to date for CMT, genes and phenotypes associated with mitochondria dynamics are summarized. Mitochondria undergo fission at the interface of the mitochondria-endoplasmic reticulum (ER) driven by interaction of several accessory factors DRP1, MFF, MID49, and DNM2 [8,9]. Fusion machinery is mediated by coordination of MFN1/2 with OPA1 in the outer and inner mitochondrial membrane, respectively. GDAP1and SLC25A46 localize to the mitochondria membrane and regulate mitochondrial fusion [10]. KIF5A and DYNC1H1 are implicated in mitochondria transport and trafficking.
EMPF1/2, encephalopathy due to defective mitochondrial and peroxisomal fission; COXPD49, combined oxidative phosphorylation deficiency 49; OPA, optic atrophy; CMT2A/2B/4A/6A/2K/2O, Charcot-Marie-Tooth disease of indicated subtype; LCCS5, lethal congenital contracture syndrome 5; CNM1, centronuclear myopathy 1; MTDPS14, mitochondrial DNA depletion syndrome 14; GADP, ganglioside-induced differentiation-associated protein-1; SPG10, spastic paraplegia 10; NEIMY, s neonatal intractable myoclonus; MRD13, mental retardation, autosomal dominant 13; SMALED1, spinal muscular atrophy, lower extremity-predominant 1.
7

## Slide 8
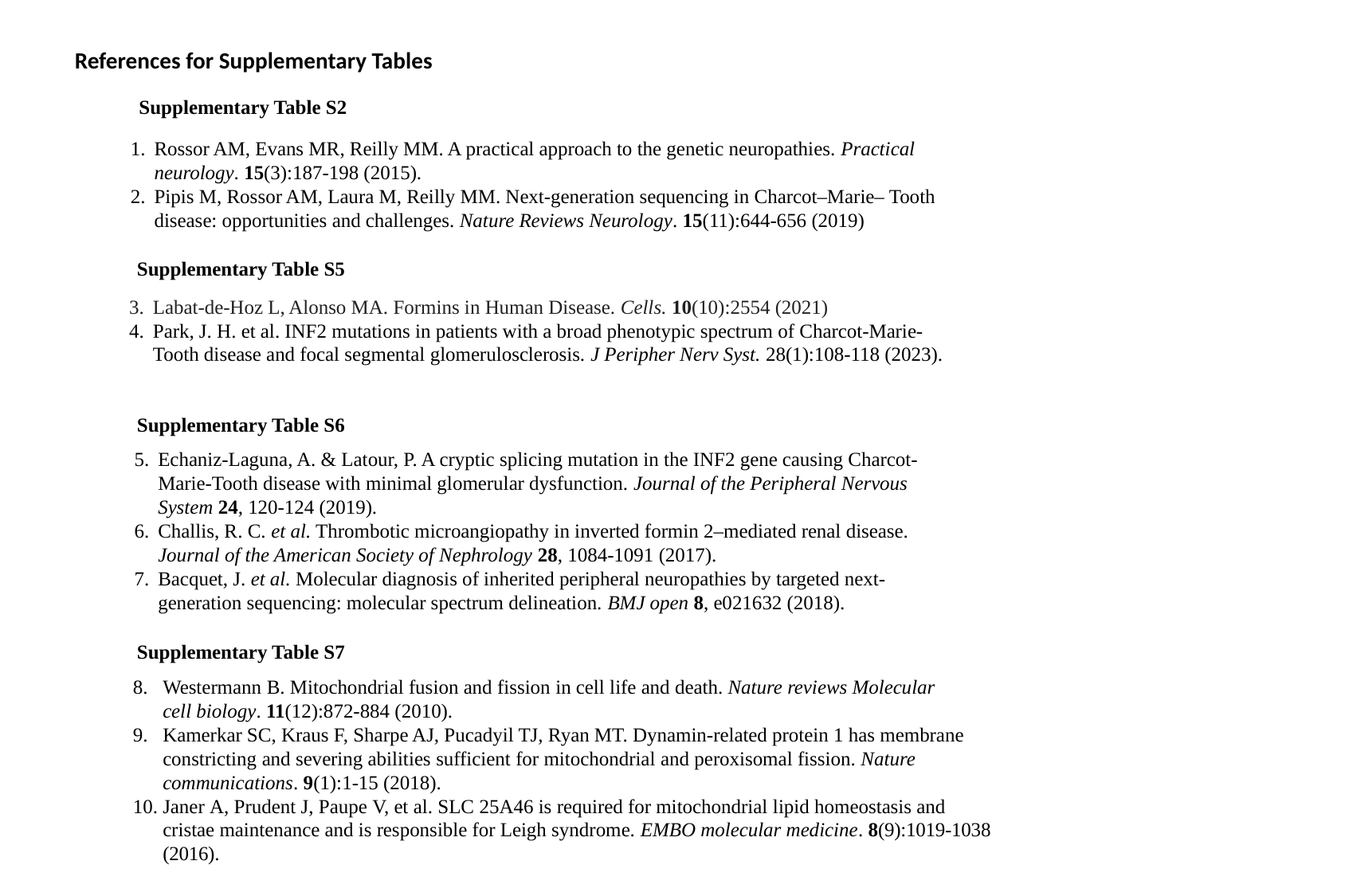

References for Supplementary Tables
Supplementary Table S2
Rossor AM, Evans MR, Reilly MM. A practical approach to the genetic neuropathies. Practical neurology. 15(3):187-198 (2015).
Pipis M, Rossor AM, Laura M, Reilly MM. Next-generation sequencing in Charcot–Marie– Tooth disease: opportunities and challenges. Nature Reviews Neurology. 15(11):644-656 (2019)
Supplementary Table S5
Labat-de-Hoz L, Alonso MA. Formins in Human Disease. Cells. 10(10):2554 (2021)
Park, J. H. et al. INF2 mutations in patients with a broad phenotypic spectrum of Charcot‐Marie‐Tooth disease and focal segmental glomerulosclerosis. J Peripher Nerv Syst. 28(1):108-118 (2023).
Supplementary Table S6
Echaniz‐Laguna, A. & Latour, P. A cryptic splicing mutation in the INF2 gene causing Charcot‐Marie‐Tooth disease with minimal glomerular dysfunction. Journal of the Peripheral Nervous System 24, 120-124 (2019).
Challis, R. C. et al. Thrombotic microangiopathy in inverted formin 2–mediated renal disease. Journal of the American Society of Nephrology 28, 1084-1091 (2017).
Bacquet, J. et al. Molecular diagnosis of inherited peripheral neuropathies by targeted next-generation sequencing: molecular spectrum delineation. BMJ open 8, e021632 (2018).
Supplementary Table S7
Westermann B. Mitochondrial fusion and fission in cell life and death. Nature reviews Molecular cell biology. 11(12):872-884 (2010).
Kamerkar SC, Kraus F, Sharpe AJ, Pucadyil TJ, Ryan MT. Dynamin-related protein 1 has membrane constricting and severing abilities sufficient for mitochondrial and peroxisomal fission. Nature communications. 9(1):1-15 (2018).
Janer A, Prudent J, Paupe V, et al. SLC 25A46 is required for mitochondrial lipid homeostasis and cristae maintenance and is responsible for Leigh syndrome. EMBO molecular medicine. 8(9):1019-1038 (2016).
8
